# High-resolution structures of a siderophore-producing cyclization domain from *Yersinia pestis* offer a refined proposal of substrate binding

**DOI:** 10.1016/j.jbc.2022.102454

**Published:** 2022-09-05

**Authors:** Andrew D. Gnann, Yuan Xia, Jess Soule, Clara Barthélemy, Jayata S. Mawani, Sarah Nzikoba Musoke, Brian M. Castellano, Edward J. Brignole, Dominique P. Frueh, Daniel P. Dowling

**Affiliations:** 1 Department of Chemistry, University of Massachusetts Boston, Boston, Massachusetts, USA; 2 Department of Biology, Massachusetts Institute of Technology, Cambridge, Massachusetts, USA; 3 Department of Biophysics and Biophysical Chemistry, Johns Hopkins University School of Medicine, Baltimore, Maryland, USA

**Keywords:** crystallography, molecular docking, natural product, coenzyme, siderophore, yersiniabactin, carrier protein, 4ʹ-phosphopantetheine, A, adenylation domain, ArCP, aryl carrier protein, C, condensation domain, CP, carrier protein, Cy, heterocyclization domain, 2HPTT, 2-hydroxyphenylthiazolinylthiazolidine, MD, molecular dynamics, NRP, nonribosomal peptide, NRPS, nonribosomal peptide synthetase, PCP, peptidyl carrier protein, PDB, Protein Data Bank, PK, polyketide, PKS, polyketide synthase, Ppant, phosphopantetheine, SCBR, substrate side chain–binding region, Ybt, yersiniabactin

## Abstract

Nonribosomal peptide synthetase heterocyclization (Cy) domains generate biologically important oxazoline/thiazoline groups found in natural products, including pharmaceuticals and virulence factors such as some siderophores. Cy domains catalyze consecutive condensation and cyclodehydration reactions, although the mechanism is unknown. To better understand Cy domain catalysis, here we report the crystal structure of the second Cy domain (Cy2) of yersiniabactin synthetase from the causative agent of the plague, *Yersinia pestis*. Our high-resolution structure of Cy2 adopts a conformation that enables exploration of interactions with the extended thiazoline-containing cyclodehydration intermediate and the acceptor carrier protein (CP) to which it is tethered. We also report complementary electrostatic interfaces between Cy2 and its donor CP that mediate donor binding. Finally, we explored domain flexibility through normal mode analysis and identified small-molecule fragment-binding sites that may inform future antibiotic design targeting Cy function. Our results suggest how CP binding may influence global Cy conformations, with consequences for active-site remodeling to facilitate the separate condensation and cyclodehydration steps as well as potential inhibitor development.

Modular, multidomain polyketide (PK) synthases (PKSs), and nonribosomal peptide (NRP) synthetases (NRPSs) produce a variety of PK and NRP natural products (1, 2, 3, 4). NRP, PK, and hybrid NRP–PK molecules display bioactivities with clear importance to humans (5, 6, 7, 8), and the modularity of NRPS and PKS systems makes them prime candidates for bioengineering (9). In addition, NRP and PK secondary metabolites are primarily produced in bacteria and, to a lesser extent, fungi, and some are virulence factors of pathogenic bacteria (10, 11, 12, 13, 14); therefore, a thorough understanding and control of NRPS and PKS catalytic machinery may afford new options for antibiotic targets (15).

NRPSs and PKSs follow an assembly line logic in which each module is responsible for activating and incorporating a specific building block. Canonical NRPS elongation modules consist of adenylation (A), condensation (C), and peptidyl carrier protein (PCP) domains. A domains activate and load specific substrates (*e.g.*, amino acids) onto PCP phosphopantetheine (Ppant) cofactors as thioesters. PCP domains from contiguous upstream and downstream modules (herein called upstream/donor and downstream/acceptor PCPs) shuttle their thioester-tethered substrates to the intervening C domain, which catalyzes their condensation to yield an elongated and intermediate peptide product attached to the acceptor PCP. The product may then act as a donor intermediate in a subsequent condensation within the next module or be released hydrolytically within the final module. Substrates and intermediates are often modified by tailoring domains, giving rise to various oxidation states (16, 17, 18, 19), methylation patterns (20, 21, 22, 23, 24, 25), and other alterations (26, 27, 28, 29). Of particular interest for the present work, many NRPSs contain a variant of C domains, the heterocyclization (Cy) domain, that converts hydroxyl- or thiol-containing peptides into heterocycles during chain elongation. For more information on the C domain family, see the 2017 review by Bloudoff and Schmeing (30).

Thiazoline and (methyl)oxazoline rings formed by Cy domains are prevalent and important for the bioactivity of many natural products (Fig. S1), a prime example being thiazol(in)e-containing siderophores such as yersiniabactin (Ybt) (31). Ybt is a virulence factor of *Yersinia* bacteria, the causative agents of the plague. Ybt is produced under iron-limiting conditions to scavenge trace metal ions from the extracellular environment, including Fe^3+^ from the host (32, 33, 34). Ybt is just one of a number of thi-/oxazol(in)e siderophores required for bacterial growth in iron-restricted environments (3, 12, 35, 36, 37), and understanding the molecular mechanisms of Cy domains may support development of a new class of antibiotics against these bacteria (38, 39, 40). Sequence diversity between Cy domains of different organisms, typically sharing <30% sequence identity, might permit development of selective inhibitors against Cy domains of pathogenic species, avoiding endogenous Cy domains of the human microbiome (41).

The biosynthesis of Ybt provides an example of a noncanonical NRPS–PKS system that entails the activity of several proteins encoded within a pathogenicity island (42) (Fig. 1). We focus on the second cyclization domain, Cy2, of the NRPS protein HMWP2. HMWP2 contains the starter module of the pathway and has the domain order ArCP–Cy1–A(E)–PCP1–Cy2–PCP2, where ArCP is an aryl CP and A(E) is an interrupted A domain embedding an unconventional 37 kDa epimerase domain (24, 29). HMWP1 contains Cy3 and PCP3 in a hybrid PKS–NRPS context. ArCP is loaded with a salicyl thioester by the standalone A domain YbtE, and the A(E) domain of HMWP2 is responsible for loading l-cysteine onto PCP1 and PCP2 (in *cis*) and PCP3 (in *trans*). The embedded E domain generates a mixture of enantiomers of the first thiazoline installed by HMWP2-Cy1, as would be derived from cyclodehydration of peptides formed with either d- or l-cysteine. Since Ybt only contains one stereoisomer, HMWP2-Cy2 is expected to provide stereospecificity. The product of HMWP2-Cy2, Ppant-tethered 2-hydroxyphenylthiazolinylthiazolidine (2HPTT), is passed at an interprotein NRPS–PKS junction between HMWP2 and HMWP1. HMWP1 combines PKS and NRPS logics to further extend and release the compound, and YbtU reduces the second heterocycle of Ybt to thiazolidine. Both NRPS and PKS systems require movements of CPs to transfer elongating molecules; however, our understanding of NRPS–PKS interfaces outside docking domain studies is limited (43). Therefore, the rich noncanonical architecture of Ybt synthetase is of great interest for exploring structure–function relationships in hybrid NRPS–PKS systems. As a first step toward modeling the NRPS to PKS interface between HMWP2 and HMWP1, we explore the HMWP2-Cy2 domain, which with its acceptor PCP (HMWP2–PCP2) forms the NRPS half of the NRPS–PKS junction.

**Figure 1:**
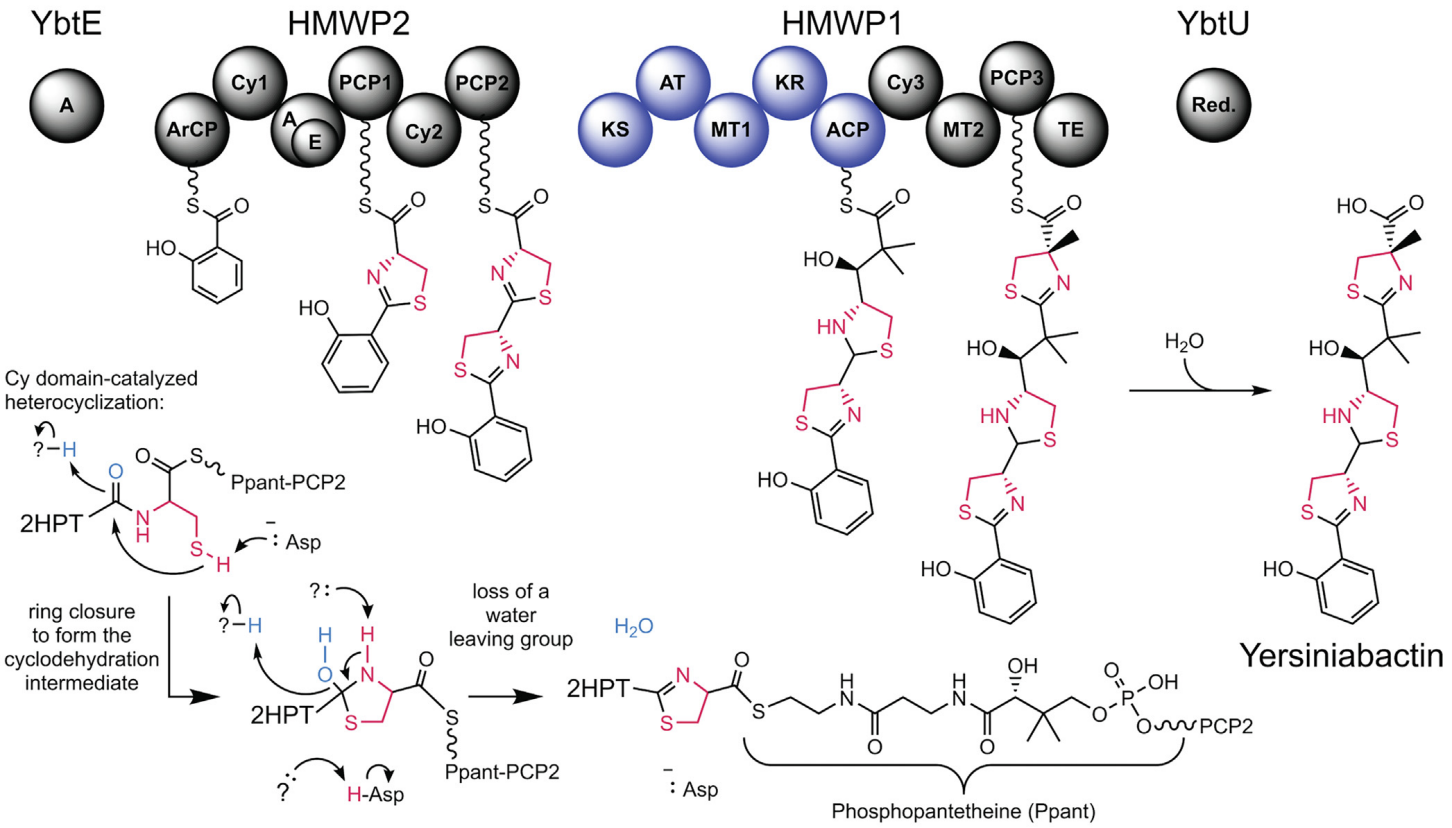
**The four protein, hybrid NRPS–PKS, modular assembly line for biosynthesis of yersiniabactin and a mechanistic proposal for HMWP2-Cy2-catalyzed heterocyclization.** Each *circle* represents either an NRPS domain (*black*) or a PKS domain (*blue*). Bond-line structures are substrates/products/intermediates carried by their respective carrier proteins. Domain labeling (*left to right*), and descriptions for PKS domains not detailed in the main text, are A, adenylation; ArCP, aryl carrier protein; Cy, heterocyclization; E, epimerization; PCP, peptidyl carrier proteins; KS, ketosynthase (catalyzes Claisen condensation of acyl donor and malonyl acceptor groups); AT, acyl transferase (catalyzes tethering of malonyl units onto the acyl carrier protein [ACP]); MT, methyltransferase (catalyzes methyl transfer from *S*-adenosyl methionine to cysteinyl or malonyl C2 positions); KR, ketoreductase (catalyzes NAD(P)H-dependent reduction of the keto group from the acyl donor substrate of the KS); ACP, acyl carrier protein; TE, thioesterase (catalyzes hydrolytic cleavage of the product thioester); red., reductase. (Not shown: YbtS, synthesizes input salicylate from chorismate; YbtD, transfers phosphopantetheine [Ppant] to CPs; YbtT, type II thioesterase putatively hydrolyzing improperly loaded substrates). The Ppant cofactor is represented as a wavy line-S and is drawn out in the *bottom right* of the figure. Atoms in *light blue* in the partial mechanism in the *bottom left* constitute the water-leaving group. Thiazoline heterocycle atoms derived from cysteine are colored *magenta* throughout the figure. NRPS, nonribosomal peptide synthetase; PKS, polyketide synthase.

NRPS Cy domains are typically responsible for both condensation and cyclodehydration reactions, affording (methyl)ox-/thiazoline modifications (Figs. 1 and S2), and their catalytic mechanisms are only partially understood. Five structures of Cy domains have been reported, involved in biosynthesis of epothilones (44), bacillamide (45), Ybt (46), JBIR-34/35 (47), and pyochelin (48). The first two Cy domain structures identified a structural role for aspartates of the characteristic DXXXXD motif, which was in keeping with the roles of corresponding residues in C domains (30, 44, 45). The Cy domain’s active site, formed in a tunnel between its N- and C-terminal subdomains, contains a putative catalytic aspartate–threonine dyad (44, 45) as well as a nearby glutamine (44), mutations of which severely affected cyclodehydration. Interestingly, the glutamine is not present in BmdB-Cy2, suggesting complementation of its yet unknown role by some other means. Several additional conserved residues affect product formation but serve unknown roles. Notably, HMWP2-Cy1 has been shown to display global dynamics, and a single mutation leads to a global molecular response (46), highlighting the limitations of traditional structural studies relying on a single static structure and the need to identify conformations critical for function. Finally, there is limited structural information for binding of (amino)acyl/peptidyl-Ppant in a Cy domain or for binding CPs in catalytically competent states, with the recent cryo-EM structures of FmoA3 (47) and PchE (48) providing the first snapshots of CP binding to Cy domains within modular contexts. Overall, more structural snapshots of Cy domains are needed to resolve important gaps in understanding how they function.

Here, we report the 1.94 Å resolution crystal structure of the Cy2 domain of Ybt synthetase (HMWP2-Cy2), which with its acceptor PCP (HMWP2–PCP2) forms the NRPS half of the NRPS–PKS interface. In addition to the role of Ybt in pathogenesis for *Yersinia pestis*, this cyclization domain is of interest because it accepts a donor that already features a heterocycle and because Cy2 is expected to govern the stereochemistry of the end product, Ybt (29). We construct protein–protein docking models suggesting interactions in the tridomain complex HMWP2–PCP1–Cy2–PCP2 contributing to the synthetic directionality of HMWP2. We then take advantage of a less constricted active-site tunnel to report covalent ligand docking experiments of intermediates during cyclodehydration tethered to PCP2 as oriented in the protein–protein docking result. The results narrow the possible positioning of a cyclodehydration intermediate, and we discuss the impact of the flexibility of HMWP2-Cy2, estimated through normal mode analysis, on positioning the condensation intermediate that precedes cyclization. The Cy conformation we present here provides important clues on communication between Cy domains and their substrates and partner CP domains, suggesting relay mechanisms relying on structural fluctuations that may couple domain engagements with active-site remodeling and global remodeling of the entire Cy domain.

## Results

### Structure of a Cy domain from siderophore biosynthesis

**Figure 2:**
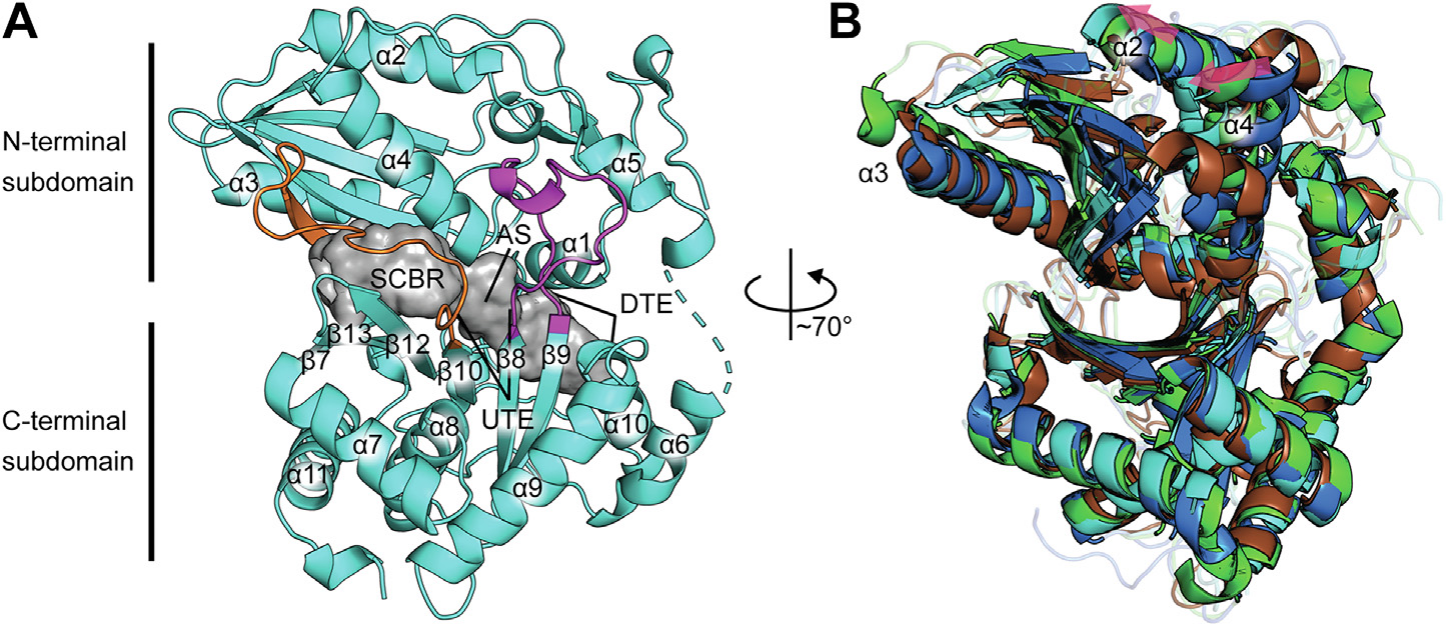
**The HMWP2-Cy2 crystal structure exhibits a more open active-site tunnel.***A*, an overview of the 1.94 Å resolution HMWP2-Cy2 crystal structure with the tunnel as a *graysurface*, the floor loop and latch in *light magenta* and *orange cartoon*, respectively, and other features discussed in the text labeled (AS, active site; DTE, downstream tunnel entrance; SCBR, side chain–binding region; UTE, upstream tunnel entrance). *B*, Cy domain C-terminal subdomain Cα alignment demonstrates intersubdomain conformational variation among Cy domains. HMWP2-Cy2 is *cyan* (PDB ID: 7JTJ), EpoB-Cy is *green* (PDB ID: 5T7Z), BmdB-Cy2 is *blue* (PDB ID: 5T3E), and PchE-Cy is *brown* (PDB ID: 7EN1). Loop regions are transparent. *Pink arrows* highlight correlated variation between the models.

Two X-ray datasets were obtained for two crystals of HMWP2-Cy2. The first dataset was solved to 1.94 Å resolution by molecular replacement using the structure of the Cy domain from epothilone biosynthesis (44) (EpoB-Cy; 35% sequence identity) as a search model. The second dataset was solved to 2.35 Å resolution using the 1.94 Å resolution HMWP2-Cy2 model (Table S1). These structures demonstrate the pseudodimeric fold characteristic of the condensation domain superfamily, which is a two-lobed arrangement of two chloramphenicol acetyltransferase–like folds (Figs. 2*A* and S3). The chloramphenicol acetyltransferase–like folds, called the N- and C-terminal subdomains here, each contain a mixed β sheet with intervening α helices. The two subdomains are linked *via* α helices 5 and 6, which resemble a hinge, with the first ∼180 residues forming the N-terminal subdomain and the remainder forming the C-terminal subdomain. The C-terminal β sheet is formed by strands 7, 13, 12, 10, 8, and 9 (in order), and the N-terminal β sheet is formed by strands 4, 5, 6, 1, and 11, with strand 11 donated from the C-terminal subdomain. Strand 11 and the preceding loop have been referred to as a latch and are thought to mediate partial dissociation of the subdomains (Fig. 2*A*) (49). The two points of crossover between the subdomains are the predominantly loop region between β-strands 8 and 9 in the C-terminal subdomain (floor loop) and the latch.

HMWP2-Cy2 crystallizes in the space group *P*4_1_2_1_2 with one molecule in the asymmetric unit. The largest interface area between Cy2 molecules within the crystal buries ∼680 Å^2^ (3.6%) of the solvent-accessible surface area, based on analysis using the PISA server (50); therefore, HMWP2-Cy2 is likely a monomer in solution, consistent with the domain’s behavior by size-exclusion chromatography (Fig. S4). Electron density maps obtained using the 1.94 Å resolution data permitted building residues Q1483–L1663 of the N-terminal subdomain and N1669–Q1910 of the C-terminal subdomain (HMWP2 numbering) but did not support building the hinge linker between the N- and C-terminal subdomains. Fortunately, the lower-resolution dataset includes weak electron density for the missing residues except P1666, which remains disordered, allowing for generation of a composite model using coordinates from the higher-resolution dataset with residues from the hinge linker derived from the 2.35 Å resolution structure. P1666 was built and refined to reduce potential energy in the final composite model and achieve acceptable connectivity. The weak or missing electron density for the connection at the hinge helices is consistent with greater flexibility in this area of HMWP2-Cy2.

Of the prior Cy domain structures, the Cy domain from EpoB (44) most closely resembles HMWP2-Cy2 (RMSD of 1.4 Å between 338 of 431 Cα atoms, aligning against Protein Data Bank ([PDB] ID: 5T7Z). Interestingly, protein alignments improve when considering either the N-terminal subdomain (residues 1483–1665) or the C-terminal subdomain (residues 1666–1910) independently, consistent with movement of these subdomains relative to each other within published NRPS Cy domain structures (Fig. 2*B*) (44, 45, 46, 47, 48). Using a Cα alignment of the C-terminal subdomains of HMWP2-Cy2 with other Cy domains depicts a twisting of the N-terminal subdomain β sheet, with concomitant repositioning of helices α2 and α4 on one side of the N-terminal subdomain β sheet and of α3 on the opposite side.

The conformation of HMWP2-Cy2 defines an open and continuous tunnel, with a solvent-accessible surface volume of ∼543 Å^3^ (Figs. 2 and S5). As discussed later, the acceptor site is open, whereas the donor-binding site appears closed off, generating a tunnel from the donor substrate side chain–binding region (SCBR) to the acceptor site. Although this tunnel has been observed in EpoB-Cy, BmdB-Cy2, and condensation domains (44, 45, 51, 52), the tunnels of EpoB-Cy and BmdB-Cy2 are more constricted (Fig. S5) with the BmdB-Cy2 tunnel returning a volume of ∼382 Å^3^ and EpoB-Cy's appearing as three chambers collectively measuring ∼199 Å^3^. In particular, the EpoB-Cy and BmdB-Cy2 structures are constricted in the vicinity of the donor substrate SCBR.

The putative catalytic dyad residues (D1862 and T1830) and helper residue (Q1864) are found in conformations resembling EpoB-Cy (44) and BmdB-Cy2 (45). Intriguingly, the HMWP2-Cy2 active site contains an unexpected metal-binding site against β1 and the latch strand. This metal site involves three backbone carbonyl groups (S1520, H1521, and Q1855), a carboxamide (N1621 following the first aspartate of the DXXXXD motif), and two crystallographic waters, giving rise to a distorted octahedral coordination geometry (Fig. S6). This site refines as a sodium with a *B*-factor of 28.1 Å^2^ compared with an average of 24.8 Å^2^ for protein atoms with which it interacts. The position after the first aspartate of the DXXXXD motif is most commonly leucine or methionine, with asparagine being the fourth most common (Fig. S7). There also does not appear to be any preference for a polar amino acid at the second position of the DXXXXD motifs within a subset of Cy domain sequences selected from siderophore NRPSs (Fig. S8).

### CP interaction surfaces

**Figure 3:**
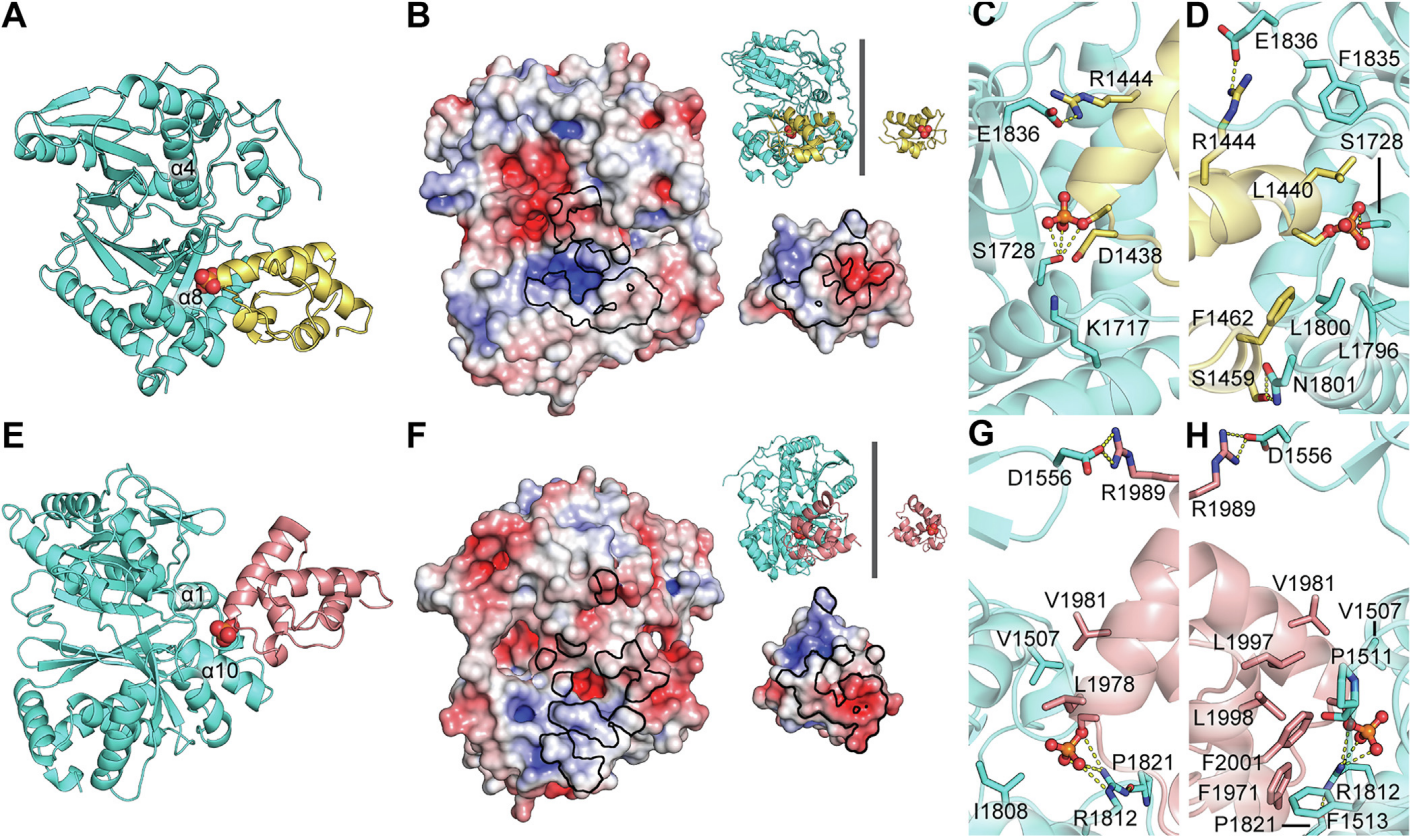
**Protein–protein docking poses of HMWP2–PCP1 and HMWP2–PCP2 with HMWP2-Cy2.***A*, a model of HMWP2–PCP1 (*yellow cartoon* with the phosphate of phosphoserine in spheres with elemental coloring) is shown bound at the expected upstream PCP-binding site of HMWP2-Cy2 (*cyan*). *B*, electrostatic potential maps of the upstream PCP-binding site of HMWP2-Cy2 (*left*) and the Cy-binding site of HMWP2–PCP1 (*right*) are related here by a rotation of 180° so that the interaction surfaces of both components are displayed. The orientations of the components can be observed in the *inset cartoon* representations. Electrostatic potential maps use a color scale from −5 to +5 k_B_T/e_c_ (*red* to *blue*)_._ The interaction footprint shared by the proteins is outlined in *black*. *C* and *D*, detail of interacting residues (*sticks*) at the PCP1–Cy2 interface (phosphoserine is displayed in *ball and sticks* here). *E*–*H*, same as *A*–*D* but for the downstream HMWP2–PCP2 interaction (PCP2 is in *salmon*).

HMWP2–PCP1 is expected to bind to Cy2 at the N terminus of α8, where the donor Ppant would extend between the C termini of β8 and β10 in the C-terminal subdomain, near the N termini of helices α4 and α8 (Fig. S9*A*) (48, 51). For the binding site to become accessible to a donor Ppant in HMWP2-Cy2, β10 would need to splay away from β8 by 2 to 3 Å relative to its position in the crystal structure (Fig. S9*B*). The NMR solution structure of HMWP2–PCP1 (53) was used to investigate PCP1–Cy2 interactions with the conformation displayed by Cy2. To better mimic a tethered Ppant, a HMWP2–PCP1 model bearing a phosphoserine at S1439 (SEP1439) was prepared and docked against HMWP2-Cy2 using HADDOCK2.4 (54, 55, 56). Guiding the phosphoserine toward the expected upstream tunnel entrance (which is closed in HMWP2-Cy2) returned clusters of poses (Figs. 3*A* and S9*A*) that resemble what is observed in linear gramicidin synthetase, LgrA-PCP1-C2 (51) and the recently reported cryo-EM PchE structure (48) (Fig. S9*A*). The surface electrostatic potential reveals a charge complementarity footprint that favors this binding mode (Fig. 3*B*). Interacting polar/charged residue pairs include (in HMWP2 numbering) E1836_Cy_–R1444_PCP_, K1717_Cy_–D1438_PCP_, and N1801_Cy_–S1459_PCP_, and there is hydrophobic packing around L1800_Cy_ and F1462_PCP_ (Fig. 3, *C* and *D*). The position of HMWP2–PCP1 permits the six-residue interdomain linker omitted in the docking calculation to reach HMWP2-Cy2 without requiring rearrangements.

At the downstream tunnel entrance, which interacts with the acceptor HMWP2–PCP2, loop 1 (after α1) extends further toward α10 than in EpoB-Cy (44) or BmdB-Cy2 (45), in which it is blocked by the loop following α10 (Fig. S10, *A*–*C*). Docking experiments similar to those conducted for HMWP2–PCP1 were conducted with a homology model of HMWP2–PCP2 built on the PCP from *Streptomyces* sp. Acta 2897 (∼34% sequence ID, PDB ID: 4PWV) (16) modified with a phosphoserine at S1977 (SEP1977). Restrained docking in HADDOCK2.4 returned clusters (Fig. 3*E*) that resemble known PCP_acceptor_ complexes (48, 52, 57, 58). Surface electrostatic potentials again reveal the footprint of charge complementarity that favors this binding mode (Fig. 3*F*). This interface involves hydrophobic packing of V1507_Cy_ against L1978_PCP_ and V1981_PCP_ and of P1511_Cy_ against L1997_PCP_, as well as stacking of the phenyl ring of the weakly conserved F1513_Cy_ with both F1971_PCP_ and F2001_PCP_ (Fig. 3, *G* and *H*). The other main contribution to HMWP2–PCP2 binding according to our model is from electrostatic interaction between a conserved arginine in α10 (R1812) and SEP1977.

Near the downstream interaction surface, two regions of interest differ in Cy domain structures reported to date. First, a conserved arginine in α1 (R1509) forms a salt bridge with the first aspartate of the DXXXXD motif in EpoB-Cy (44) and PchE-Cy1 (48) but is oriented away from this residue in HMWP2-Cy2 (Fig. S10). In BmdB-Cy2 (45), the arginine adopts an intermediate position. Similarly, a conserved glutamine in the loop after the latch (Q1858) forms hydrogen bonds to loop 1 in HMWP2-Cy2 but projects its side chain into the tunnel in EpoB-Cy (and PchE-Cy1), whereas it adopts an intermediate position in BmdB-Cy2 (Figs. S10 and S11). Positioning of R1509 and Q1858 may provide a link between binding of PCP2, remodeling of its binding site, and remodeling of the active-site tunnel for catalysis in the presence of substrates.

### Exploration of cyclodehydration intermediate binding in HMWP2-Cy2 highlights potential roles for active-site tunnel residues

**Figure 4:**
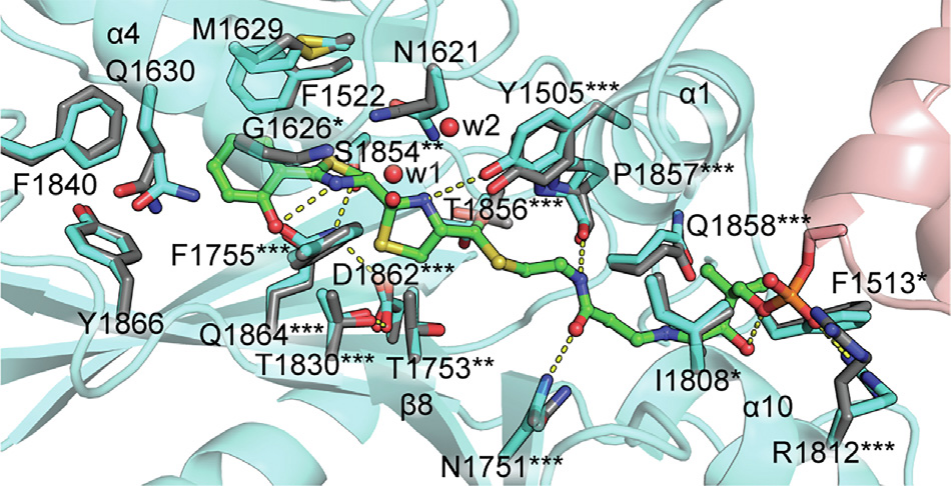
**The HMWP2-Cy2 crystal structure acceptor tunnel and active site accommodate a cyclodehydration intermediate.** Covalent docking of Ppant-2HPTT-OH in an HMWP2-Cy2–PCP2 complex based on the Cy2 crystal structure and PCP placement from protein–protein docking supports a model in which catalysis proceeds through an *R* hydroxythiazolidine intermediate (*green*) in HMWP2-Cy2 (refined covalent docking Cy model in *cyan* and PCP in *salmon* with transparent *cartoon*, and crystal structure coordinates for displayed residues in *gray*). This pose (#10 in Fig. S16) is selected as a top candidate based on its Glide docking score from covalent docking, MM-GBSA–approximated binding energy for the corresponding noncovalent phosphonate–S1977A complex and ligand and receptor strain energies. Two crystallographic water sites (*red spheres*) are shown in the vicinity of the active site (w1 and w2). HMWP2 residues marked with *asterisks* agree with Cy domain conservation trends at positions > ca. 2 bits in the sequence logo (Fig. S7) that are somewhat conserved (∗property conserved or HMWP2-Cy2 residue in at least ∼25% of aligned sequences), moderately conserved (∗∗primarily two options and HMWP2-Cy2 residue at least ∼50%), or highly conserved (∗∗∗overwhelmingly one option). MM-GBSA, molecular mechanics with generalized Born and surface area; PCP, peptidyl carrier protein; Ppant, phosphopantetheine.

We sought to determine whether the active-site tunnel of the HMWP2-Cy2 crystal structure could accommodate its cognate cyclodehydration intermediate, following cyclization but preceding dehydration. Because nucleophilic attack during cyclization can occur on either face of the condensation product amide, we employed rigid receptor covalent docking to sample binding modes of both 2*R* and 2*S* diastereomers of the intermediate mimic (2*R/S*,4*R*)-2,2-hydroxy-(2ʹ-(2ʺ-hydroxyphenyl)-thiazolinyl)-thiazolidinyl Ppant (Ppant-2HPTT-OH) (Fig. S12). The covalent docking approach permits identification of docked poses that are compatible with covalent linkage to the conserved serine of our HMWP2-Cy2–PCP2 complex (SEP1977). Both diastereomers of the hydroxythiazolidine moiety docked with favorable Glide scores in the HMWP2-Cy2 tunnel, returning in total 120 poses, which were then refined. Models of top-ranked poses obtained by this approach are displayed in Figs. S13–S18. These poses can be organized into three categories based on the direction in which the leaving group oxygen of the hydroxythiazolidine moiety is directed: (1) hydroxyl roughly toward the N-terminal end of helix α4, (2) hydroxyl toward the N-terminal subdomain β sheet, or (3) hydroxyl roughly toward the putative catalytic dyad. Depending on the chirality, the other reactive groups of the intermediate, namely the thiazolidine ring N–H and S, interact with alternative regions of Cy2. Tests with both protonation states of the active-site dyad favored the neutral form (presumed after cyclization) for both diastereomers. Figure 4 shows the top pose for the *R* intermediate, which directs the leaving group oxygen (previously of the carbonyl electrophile) toward the N terminus of helix α1. Excitingly, the top *R* intermediate pose places the hydroxythiazolidine moiety between the positions expected for the nucleophile and electrophile based on existing structures (51, 57, 59) (Fig. S19), and the placement agrees well with the product-bound structure reported for PchE-Cy1 (48) (Fig. S20).

Although comparable docking metrics were obtained for the top *R* and *S* poses, the *R* hydroxyl-toward-α4 binding mode warrants further consideration for several reasons. First, in one category of poses, the leaving group oxygen is directed roughly toward the partial positive charge of the N terminus of α4. This position is more compatible with expectations based on trapped condensation states (51) and with the cyclodehydration model proposed by Bloudoff *et al*. (45), although the leaving group oxygen is directed away from T1830, rather than toward it. This set of poses places the ring sulfur in the middle of the catalytic dyad and the ring nitrogen such that it could form hydrogen bonds with the hydroxyls of Y1505 and T1856. The Ppant arm interacts with N1751, P1857, and Q1858, and its dimethyl group is rotated toward the loops following helices α1 and α10. Alternative poses suggest that T1856 could interact with the Ppant-2HPTT-OH thioester. Two crystallographic water molecules found in the active site between helix α4, the DXXXXD motif loop, and Y1505 were found to be roughly compatible with Ppant-2HPTT-(*R*)OH positioning, although the water molecule nearest helix α4 might instead resemble positioning of the water-leaving group. Additional observations within the full set of docking results, including descriptions of possible donor side-chain interactions, are presented in the *Supporting Results* section of the Supporting information document.

### Low-frequency vibrational normal mode analysis for HMWP2-Cy2

Because Cy domain crystal structures without donor CPs display closed conformations that preclude donor substrate binding, we explored the flexibility of HMWP2-Cy2 around its putative cyclodehydration-competent conformation using normal mode analysis. Although our top cyclodehydration intermediate candidate pose is promising, it is also interesting to consider how flexibility of the Cy domain alters relationships between groups within the active site and remodels donor and acceptor CP-binding sites.

The 10 lowest-frequency normal modes of the HMWP2-Cy2 conformation were calculated using a model supplemented with water molecules to fill voids (primarily in the tunnel). The observed modes involve relative motions of the N- and C-terminal subdomains and affect the shape of upstream and downstream entrances to different extents (see movies in the Supporting information section). This flexibility is reminiscent of variations among crystal structures. Strikingly, rearrangements of both tunnel entrances co-occur across the calculated modes, supporting the idea that remodeling of one entrance upon CP binding may be coupled with global conformational change affecting the other entrance.

Mode 5 is a highly localized motion involving R1817 at the C terminus of α10 at the downstream opening. R1817 forms a salt bridge with D1679 in α6, following the hinge region that is disordered in the HMWP2-Cy2 crystal structures. Although mode 5 does not directly involve this hinge, the hinge undergoes motion in all the other modes. Higher-frequency modes 7–10 feature more motions within subdomains, especially between two lobes of the C-terminal subdomain consisting of (1) the floor loop, the strands preceding and following it (β8/9) and helices α6, 9, and 10; and (2) strands β7, 10, 12, and 13 and helices α7, 8, and 11. We note that mode 7 resembles an amplified combination of the lower-frequency modes in that rotations of N- and C-terminal subdomains result in modulation of the α3–α11 distance and displacement of α10 relative to α1 (Fig. S21). These motions result in opening of the upstream tunnel entrance by over 3 Å, which would accommodate donor Ppant binding.

**Figure 5:**
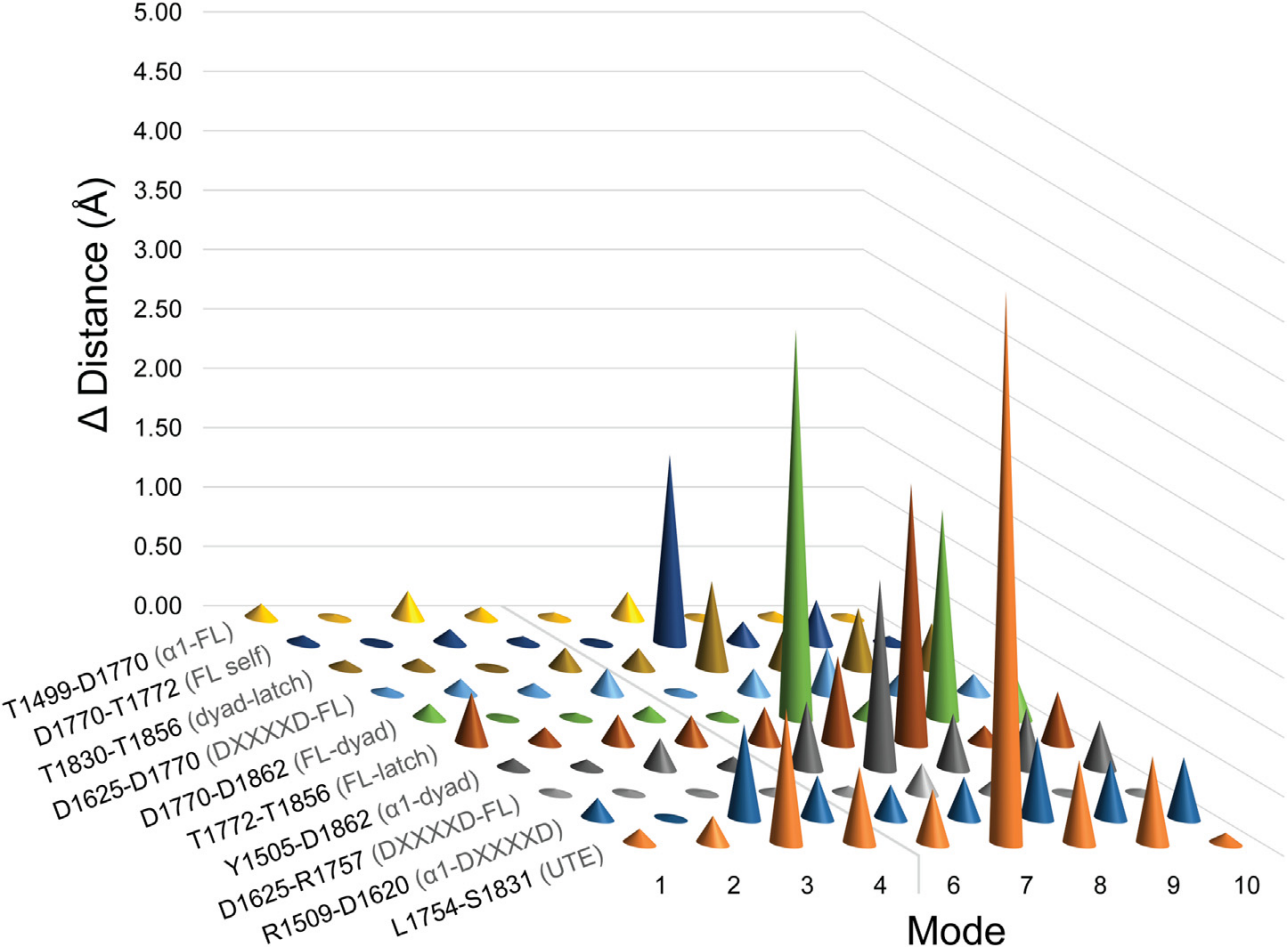
**Change ininter-residue distances with the top 10 low-frequency normal vibrational modes of HMWP2-Cy2.** A model of HMWP2-Cy2 returns a variety of motions in the normal mode analysis, primarily involving changes of orientation between the N- and C-terminal subdomains. Marked in the rows of this plot are inter-residue distances relevant for considerations of active-site shape and/or the structure of PCP/Ppant-binding regions. See Figures 4, S9*B*, and S10*C* for the structural contexts of these residues. The *light gray line* separating modes 4 and 6 indicates omission of mode 5, which involved only side-chain motion of R1817 and did not influence the measurements presented here. PCP, peptidyl carrier protein; Ppant, phosphopantetheine.

We next considered relative motions of residues in the first and second shells of the active site (Fig. 5). Several modes demonstrate modulation of the active-site dimensions in ways that would influence substrate binding. As mentioned previously, many of the modes influence access to the upstream tunnel entrance, measured by displacement of L1754 and S1831. Modes 1, 7, 8, and 10 modulate width along an axis between the floor loop and the latch strand (T1772–T1856). Modes 7 and 9 modulate the distance between the conserved aspartate of the floor loop (D1770) and the active-site aspartate (D1862), as well as the distance between the dyad threonine (T1830) and T1856 in the latch. Modes 6 to 10 appreciably modulate the distance between Y1505, which interacts with the substrate in the acceptor tunnel, and the active-site aspartate.

These modes involve motions associated with the transition between open-for-condensation and closed-for-cyclodehydration conformations, bringing the catalytic dyad toward and away from Y1505. These observations, when considered alongside our top *R* cyclodehydration intermediate pose, suggest that global motion may couple to substrate rearrangements after condensation to obtain a cyclization reaction complex. Notably, the flexibility predicted by this analysis occurs without substantial displacement of the salt bridge D1625–R1757 flanking the upstream entrance, which is consistent with a PCP–C interface from LgrA (PDB ID: 6MFX) (51) but contrasts the recent PchE-ArCP–Cy1 cryo-EM model, which shows this salt bridge broken in the CP–Cy complex presumed to resemble the open-for-condensation state. The other salt bridge between R1509 and D1620, linking the DXXXXD motif to helix α1, does undergo slight displacements in modes 3 to 10, which is reminiscent of variation at this position in the Cy crystal structures. The other interesting interaction with α1, the hydrogen bond between T1499 and D1770 in the floor loop, is relatively consistent across the modes. How these networks of interactions are maintained or broken across the reaction cycle of Cy domains will be interesting, as modulation of their environments by CP binding or chemical changes in the active site may be key for productive trajectories through the conformational landscape.

### Potential inhibitory hot spots for HMWP2-Cy2

**Figure 6:**
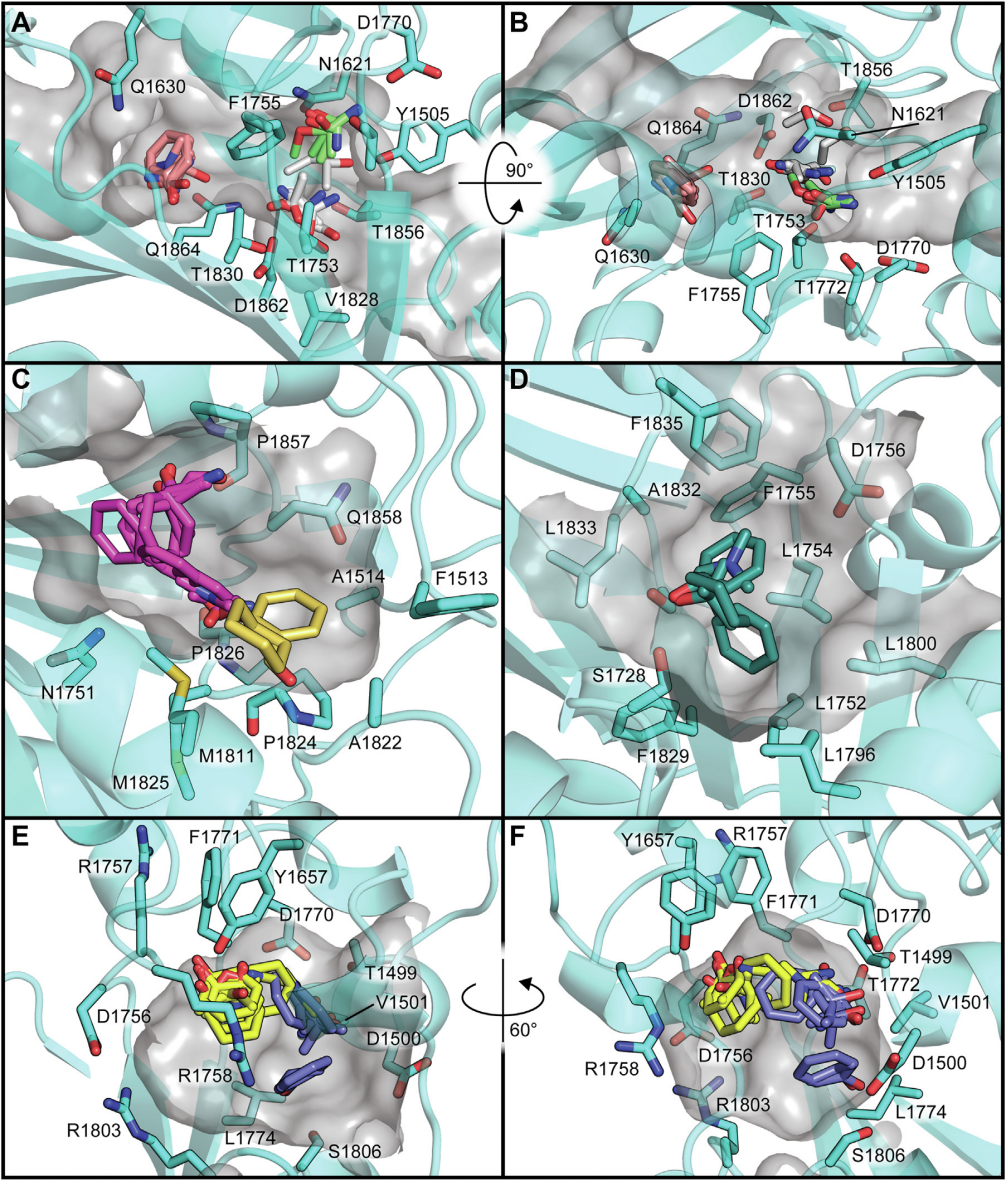
**FTmap server results for HMWP2-Cy2.***A* and *B*, two views of small ligand clusters in the HMWP2-Cy2 active site are shown related by a 90° rotation around the *X*-axis. *C*, two clusters of small organic ligands in the downstream tunnel are shown. *D*, binding of a cluster of small organic ligands at the upstream tunnel entrance relies largely on hydrophobic packing, but specific polar contacts could also occur with S1728, which marks the expected phosphopantetheine phosphate–binding site at the end of helix α8. *E* and *F*, two clusters of small organic ligands bind in a cavity between the floor loop, hinge, and helices α1 and α9. Although some polar contacts are observed between protein and members of this cluster, docking at this site is probably largely driven by burying hydrophobic surface area.

To identify locations within the HMWP2-Cy2 structure for future inhibitor development, we analyzed the structure with the FTMap server, which returns sites for a set of chemical probes (Fig. 6) (60). Several hot spots were identified within the protein tunnel (Fig. 6, *A*–*C*) and may guide designs of competitive inhibitors mimicking the Ppant-tethered intermediates. Notably, a cluster in the SCBR favors ring structures, including phenols, and roughly mimics the position and orientation of the 2-hydroxyphenyl-thiazolinyl moiety in our docking models for the *R* intermediate pose (Fig. S22). Clusters near the N terminus of helix α4 or next to the active-site dyad place polar groups in orientations consistent with positions of polar groups in the intermediate docking results. In contrast, the Ppant-binding region does not return clusters exhibiting specific polar contacts, but probes are found engaged in hydrophobic interactions. Excitingly, three hot spots were identified outside the protein tunnel: a minor site located at the expected donor-binding site (Fig. 6*D*), and two more populated sites nestled between the floor loop and α1 (Fig. 6, *E* and *F*). Both sites seem driven by hydrophobic packing, but the donor site also features polar contacts with S1728 at the predicted Ppant phosphate-binding site. Therefore, in addition to competitive inhibitors mimicking intermediates, alternative classes of inhibitors may be developed to bind PCP sites or the floor loop. The floor loop has been shown to undergo fluctuations in surface electrostatic potential across the conformational variation of the C domain family (46) despite divergent sequences. Blocking key conformational transitions for a specific Cy domain may permit targeting specific organisms.

## Discussion

The crystallographic structures of HMWP2-Cy2 presented here add to the limited array of structural information about Cy domains, and the docking studies and normal mode analysis performed here suggest how malleability may be related to distinct steps of catalysis. What seems clear is that Cy domains prefer closed upstream entrances in the absence of PCP interactions, so introduction of a PCP may be required to shift the conformational equilibrium toward an open-for-condensation–like state, as observed in cryo-EM studies of PchE (48). In the absence of PCPs, obstruction of the donor site comes in part from a conserved phenylalanine at the C-terminal end of β8 leading into the floor loop (HMWP2 F1755), as seen in crystallographic studies. Mutation of this residue was found to decrease cyclodehydration activity severely (45, 48). Jointly, these observations form the basis for the suggestion that Cy domains exclude bulk solvent from the cyclodehydration active site to facilitate the dehydration step (45). In the presence of a donor PCP, this phenylalanine residue rotates away from the end of helix α4 (48), and it might be that early recognition events between the Cy domain and the PCPs alter the conformational preferences of the phenylalanine and nearby residues. Among these residues, T1753 and T1830 flank the upstream opening and would sense the presence of donor substrates. However, T1830 is also part of the putative catalytic dyad and interacts with the acceptor substrate and D1862. It is likely then that breaking or forming interactions with T1753 and T1830 would influence the global conformational change between open and closed states as well as chemical properties of the catalytic dyad. In some of our docking models, T1753 is nearly able to interact with the sulfur of the acceptor Ppant thioester linkage and is primed to induce such conformational changes. A malleable cyclization scaffold is in agreement with the aforementioned solution NMR studies (46), which revealed global dynamics in the cyclization domain under study, and with cryo-EM data of NRPS modules (47, 48) where cyclization domains display lower resolutions than other domains. The solution NMR studies also indicate that the conserved and putative catalytic aspartate plays a key role in global dynamics that mediate allosteric communication of CP binding (46), which works with our model to suggest how PCP binding, global conformation, and the stage of catalysis may be related.

**Figure 7:**
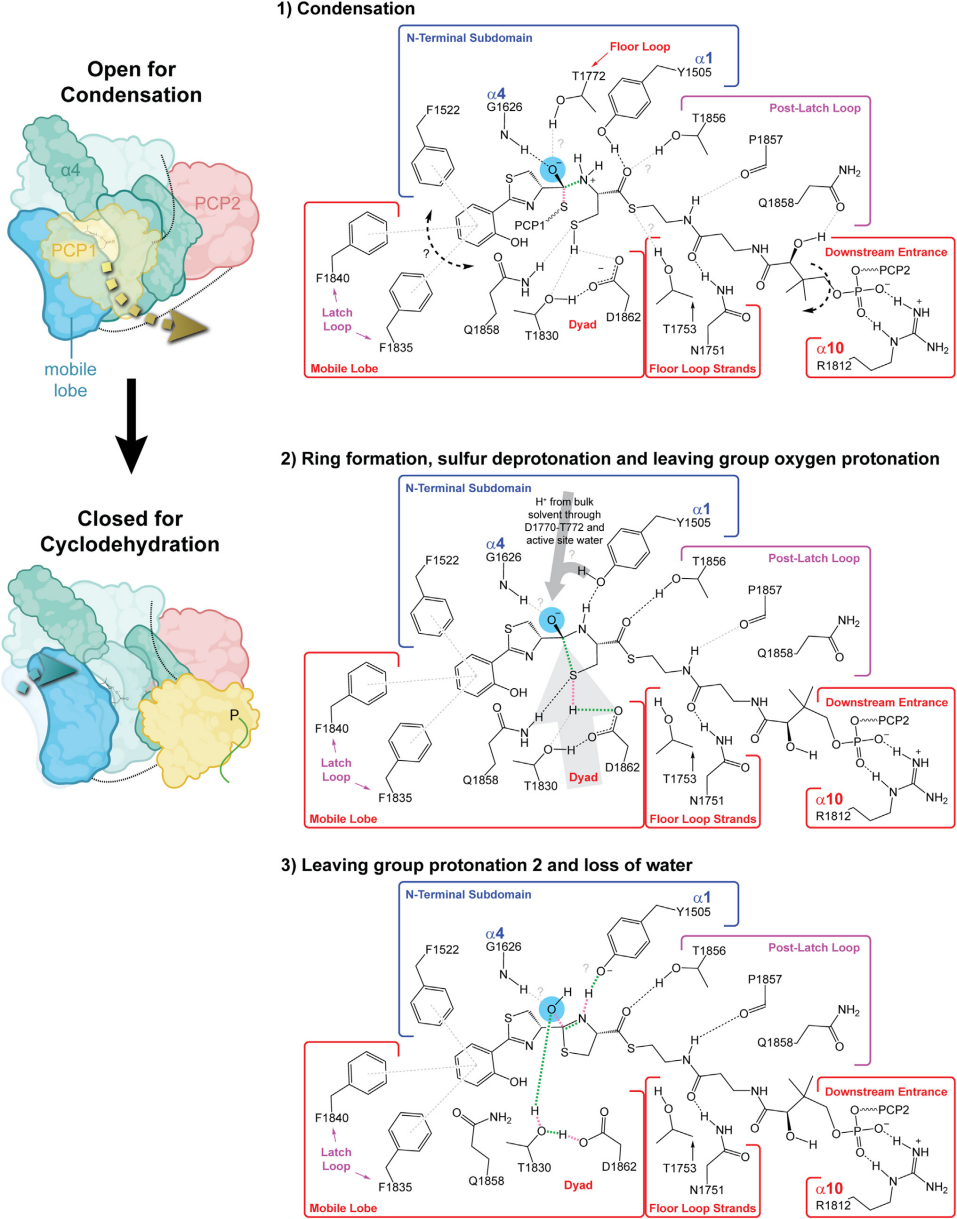
**Proposals for a global Cy domain conformational change associated with catalysis and three main reaction steps.** On the *left* is a schematic representation of the conformational changes involved in transitioning from the open-for-condensation state to the closed-for-cyclodehydration state (created using BioRender). The PCP_donor_ (*yellow*) dissociates from its position in the open-for-condensation state (*yellow arrow*, Cy domain in *green/blue*) at least enough to remove the pantetheine moiety from the upstream tunnel entrance. Helix α4 is labeled for reference, and other *darker green regions* in the open-for-condensation state are the floor loop and hinge regions. To obtain the closed-for-cyclodehydration state, one lobe of the C-terminal subdomain (*blue*, mobile lobe), containing T1830 and D1862, rotates through 5 to 10° around its point of contact with the floor loop strands (*teal arrow*). In addition, a gating phenylalanine (HMWP2-Cy2 F1755) in the floor loop swings toward the N terminus of helix α4 (not depicted). On the *right* are proposals for active-site organization during three main reaction steps. *Green* and *pink dashed lines* indicate bonds formed or broken in each step, respectively. *Light gray dashed lines* indicate interactions suggested by our model but for which there is less confidence given variation in our docking models. The first step, condensation, occurs in the open-for-condensation state of the Cy domain. *Gray question marks* in step 1 point out potential interactions between residues around the active site and the Ppant thioester or oxyanion. In the state depicted in step 1, it appears possible that the donor side chain could form a range of interactions with hydrophobic residues around the SCBR (symbolized by the *bold*, *black*, *dashed arrow*, and *question mark* on the *left*). Deprotonation of the zwitterionic intermediate may proceed *via* the thiolate of the departing donor Ppant (not shown). Following displacement of the donor Ppant and PCP after condensation, an acceptor Ppant conformational change characterized by rotation of the dimethyl group by ∼180° may be concomitant with the open-to-closed transition (*bold*, *black*, and *dashed arrow* on the *right*). The second step, in which the condensation product collapses into a cyclization reaction complex, may accompany motion of the C-terminal β strands harboring the dyad (*large*, *light-gray arrow*) during the transition from the open-for-condensation state to the closed-for-cyclodehydration state. Motion of the dyad could contribute to compression of the nucleophile–electrophile distance and proton abstraction from the nucleophile. Formation of the cyclodehydration active site during the open-to-closed transition would be influenced by the states of the gating F1755 (omitted for clarity) and floor loop. The final step, including loss of a water, is envisioned as happening entirely in the closed-for-cyclodehydration state. *Gray question marks* in steps 2 and 3 point out remaining ambiguities. PCP, peptidyl carrier protein; Ppant, phosphopantetheine; SCBR; substrate side chain–binding region.

In comparison to C domains, there are additional catalytic demands for the cyclization reaction (Fig. 7): the geometry of the condensation intermediate must be controlled to overcome ring strain when forming the hydroxythiazolidine intermediate and to allow efficient proton transfer for dehydration. All the Cy domains (44, 45, 46, 48) share features of the downstream tunnel that could contribute to binding the Ppant moiety in the reported manner, and these interactions are distinct from those in C–PCP models (51, 52, 58). Structural alignments with existent structures of C–PCP_acceptor_ complexes harboring Ppant molecules demonstrate that Ppant conformations in those models are inconsistent with accommodation of a cyclodehydration intermediate in the Cy active site. This adds some credence to the possibility of separate enzyme conformations for the condensation and cyclodehydration intermediates. Collectively, the Cy domain structures to date, structural comparison with prior PCP_donor_–C and C–PCP_acceptor_ complexes, and molecular docking work presented here lead us to propose that conserved residues of the downstream tunnels in Cy domains, thought to be involved in Ppant binding, may dynamically contribute to the energy landscape of an open-to-closed transition involving translation of the substrate between condensation- and cyclodehydration-competent conformations. Translation along that path could be facilitated by rearrangement of T1753 and the Q1858 and N1751 carboxamides and by the ability of Y1505 to act as either hydrogen bond donor or acceptor to the Ppant-linking thioester or the newly formed hydroxythiazolidine’s nitrogen. In addition, we show how the distinct structure of Cy domains at the downstream tunnel provides a basin of attraction that could support rearrangement of the Ppant dimethyl moiety during the open-to-closed transition. Perhaps these features of Cy domains could be targeted in inhibitor design, especially considering the identification of ligand-binding hot spots in the downstream tunnel.

In the open-for-condensation state, the putative catalytic dyad would be shifted away from the opposing side of the active site (formed in part by Y1505 in helix α1). This would open additional tunnel volume for binding the cysteine thiol near the dyad in the event of initial condensation by the amine nucleophile. Our studies support this positioning and imply an amine-first condensation step. Envisioning extending the C–S bond formed in the cyclization reaction from its length in our docked cyclodehydration intermediate model to a precyclization state reveals the potential for change in this bond-forming coordinate to be accompanied by a large-scale conformational change between open and closed states approximated in our low-frequency normal mode analysis. The conformational change we describe brings the putative catalytic dyad closer to the center of the active site, potentially positioning it to act as a base while compressing the C–S axis for bond formation (Fig. 7, step 2). Binding of the cyclodehydration intermediate in the manner we report would permit sequestration of the water-leaving group near the N terminus of α4 (which holds water in the crystal structure) and deprotonation of the ring nitrogen by the mostly conserved Y1505.

The results presented here contribute toward deciphering the complex behavior of Cy domains and present possible sites for inhibitor development targeting Cy domain activity in a crucial enzyme of a problem pathogen.

## Experimental procedures

### Cloning and expression of HMWP2-Cy2

The encoding sequence for HMWP2-Cy2 was amplified from an expression vector containing the entire HMWP2 gene, kindly provided by the C.T. Walsh Laboratory (61). The sequence encoding P1480–Q1910 was amplified using Pfu Ultra II (Agilent) with primers purchased from IDT (forward primer: 5′-GGA AAA CCC A/TA TGC CGG TCG AAC AAC-3′; reverse primer: 5′-CGG/GAT CCT ATT GCC AGG CGC TTT CAT CC-3ʹ; NdeI and BamHI binding sites are underlined, and / marks the cut site). Reactions were purified by electrophoresis and extracted using a Qiagen Gel Extraction kit. Subsequently, both pET28a (Novagen) and the recovered HMWP2-Cy2 DNA insert were double digested with NdeI and BamHI (NEB). The digested pET28a was further reacted with shrimp alkaline phosphatase (NEB) overnight at 16 °C. Digestion reactions were purified using a Qiagen Spin Clear and Concentration kit, and resultant DNA was used for ligation reactions with T4 DNA Ligase (NEB). Ligated product was transformed directly into chemically competent NEB5α cells (NEB) and plated on Miller lysogeny broth/agar solid medium supplemented with 50 μg/ml kanamycin. Transformant DNA was isolated and sequenced (Genewiz) to confirm the presence of the intact HMWP2-Cy2 DNA sequence.

The sequenced HMWP2-Cy2 construct was transformed into BL21(DE3) cells (Stratagene) for protein expression and purification. Eight 1 l lysogeny broth–Miller cultures supplemented with 50 μg/ml kanamycin were inoculated at 16 °C with 5 ml of an overnight culture and allowed to grow for approximately 72 h. Cells were harvested at 5000 rpm for 15 min in a JLA 9.1000 Beckman rotor at 4 °C. Cell pellets were immediately resuspended in approximately 40 ml of 50 mM Tris (pH 8.0), 100 mM NaCl, 5 mM β-mercaptoethanol, and 5 mM imidazole. The cell suspension was then sonicated using a Branson Sonifier at 50% amplitude and 1 s pulse/2 s off for a total of 2 min for six cycles. The cell lysate was then centrifuged for 1 h at 15,000 rpm in a JA 25.50 rotor at 4 °C. The resulting supernatant, including the soluble HMWP2-Cy2 protein, was further purified at 4 °C.

The HMWP2-Cy2 supernatant was loaded at 1 ml/min onto a 5 ml HisTrap column (GE Healthcare) pre-equilibrated with lysis buffer. Protein was eluted using a 100 ml linear gradient from 0 to 300 mM imidazole. The purest protein-containing fractions as analyzed by SDS-PAGE were collected and dialyzed into 50 mM Tris (pH 8.0) with 5 mM DTT. Dialyzed sample was then loaded onto a 10 ml Mono Q column (GE Healthcare) pre-equilibrated in the same buffer, and protein was eluted over a linear gradient from 0 to 500 mM NaCl. Protein fractions were pooled, concentrated, and run on a 16/600 S200 column (GE Healthcare) pre-equilibrated in 50 mM Tris (pH 8.0), 100 mM NaCl, and 5 mM DTT. Protein-containing fractions were collected and concentrated to 10 mg/ml as determined by the Bradford method with standard bovine serum albumin. Protein was aliquoted, flash frozen in liquid nitrogen, and stored at −80 °C. Analytical gel filtration was performed with an ENrich SEC 650 10 × 300 column (Bio-Rad), equilibrated and eluted with 50 mM Tris pH 8.0, 100 mM NaCl, and 1 mM DTT at a flow rate of 1 ml/min. Protein standards were from Sigma–Aldrich (ovalbumin, bovine serum albumin, and lysozyme) or MP Biomedicals (bovine γ-globulins) and run in 50 mM Tris (pH 6.8) and 150 mM NaCl at 1 ml/min.

### Structure determination

#### Crystallization of HMWP2-Cy2

Conditions for crystallizing HMWP2-Cy2 were identified from sparse matrix screening using a Phoenix micropipetting robot (Art Robbins). Prepared trays were stored and monitored at 4 °C within a Rock Imager 1000 (Formulatrix). Drops of 450 nl of 10 mg/ml protein in size-exclusion chromatography buffer and 150 nl of MCSG-1 H10 (Anatrace; 0.1 M HEPES [pH 7.5] and 25% [w/v] PEG 3350) yielded singular crystals of diffraction quality. Crystals were cryoprotected with crystallization solution containing 15% (v/v) glycerol, mounted on a nylon loop, and flash cooled in liquid nitrogen.

#### Data collection, phasing, and refinement

Diffraction data for the 1.94 Å resolution HMWP2-Cy2 structure were collected at the MIT crystallization facility on a rotating copper anode X-ray generator (Micromax 007) using 1.0° oscillations. Data for the 2.35 Å resolution HMWP2-Cy2 structure were collected at a wavelength of 0.9791 Å at 100 K at the Advanced Photon Source, on beamline 24-ID-E, using 0.5° oscillations. Data were indexed to space group *P*4_1_2_1_2 using HKL2000 (62), and data statistics are presented in Table S1.

Phases for the 1.94 Å resolution dataset were solved by molecular replacement in PHASER (63, 64, 65) using the structure of EpoB-Cy (PDB ID: 5T7Z) (44), minus residues M1–S42 of the NRPS docking domain, as a search probe. The model was converted to the expected sequence of HMWP2-Cy2 with truncated side chains using CHAINSAW (66) within the CCP4 suite (67), which yielded a PHASER solution with overall LLG and TFZ scores of 128 and 13.2, respectively. Initial electron density maps were interpretable and enabled direct building of the structure. Iterative rounds of model building were carried out in Coot software (68, 69), followed by refinement in PHENIX (64, 65, 70). Initial rounds of refinement included simulated annealing, energy minimization, and *B*-factor refinements. As the model refinement converged, only energy minimization and *B*-factor refinements were performed. After building the protein chain, a continuous region of electron density was observed near R1703 of the V-shaped opening between β7 and β11, just beyond the donor substrate SCBR. Attempts to refine a chain of water molecules in this density returned positive difference electron density that suggested the presence of a polymer. Therefore, a fragment of a PEG polymer from the crystallization solution and cryoprotectant was modeled and refined with reasonable *B*-factor values and electron density. In addition to this truncated PEG molecule, waters, and a sodium ion were placed and refined into electron density. Final rounds of refinement included occupancy refinement for alternate side-chain confirmations, in addition to translation/libration/screw refinement using optimal group definitions determined within PHENIX. The final model was verified using composite omit maps and Ramachandran angle analysis using MolProbity (71), and no residues were found in disallowed regions. The final model contained all except 24 residues from the N terminus, which included the hexahistidine tag, and residues 1663 to 1668 of the loop connecting the N- and C-terminal halves of the Cy domain structure. Refinement statistics are presented in Table S1.

The 2.35 Å resolution HMWP2-Cy2 structure was solved by molecular replacement using the 1.94 Å resolution structure as a search model in PHASER through PHENIX (63, 64, 65). Rounds of model building and refinement were performed identically as aforementioned until the structure refinement converged. The final model was verified using composite omit maps and Ramachandran angle analysis using MolProbity (71) and contained all except 24 residues from the N terminus, which included the hexahistidine tag, and P1666.

### Model preparation for molecular docking

#### Cyclization domain

A complete HMWP2-Cy2 model was generated by combining residues 1483 to 1662 and 1669 to 1910 from the 1.94 Å resolution structure with residues 1663 to 1668 of the 2.35 Å resolution structure (keeping a nonredundant set of ordered water molecules from both structures). The model was prepared with neutral -CHO and -NH_2_ termini using standard methods in the BioLuminate Protein Preparation Wizard (72, 73, 74): missing atoms were built in, charges were predicted using Epik (Schrödinger) (74, 76), expected bond orders were verified, and hydrogen-bonding networks were optimized. Hydrogen coordinates were refined using the OPLS3e forcefield in the program Impact within the BioLuminate Protein Preparation Wizard (72, 73, 74, 77, 78, 79, 80). All nonwater ligands were removed from Cy domain models before preparation and simulations. Crystallographic water molecules were removed from the HMWP2-Cy2 models generated for molecular docking after preparation steps (see the Cyclodehydration intermediates subsection for further information on HMWP2-Cy2–PCP2 models for covalent docking). The HMWP2-Cy2 model used for protein–protein docking was modified by altering the rotameric state of R1812 so that its guanidinium group was toward a location similar to the guanidinium group found interacting with the pantetheine phosphate group in most C–PCP complexes (51, 52, 57). The modified R1812 rotamer was installed in a model with crystallographic waters, which was then subjected to hydrogen-bond network optimization and hydrogen coordinate refinement before water molecules were removed and R1812 only was subjected to Prime (81, 82, 83) energy minimization using the automatic method (up to two iterations of 65 minimization steps using conjugate gradient descent for large gradients and truncated Newton otherwise). HMWP2-Cy2 models for protein–protein docking ultimately included a set containing possible combinations of crystallographic rotameric states of F1513 and R1812 and the C–PCP-mimicking R1812 rotamer.

#### CPs

The first model in the deposited NMR bundle for apo HMWP2–PCP1, PDB ID: 5U3H (53), was used for docking experiments. HMWP2 residues 1404 to 1476—comprising the folded core of the apo/holo conformation of PCP1—were used. This model excludes much of the flanking and flexible linker regions. In addition, the conserved serine was phosphorylated to approximate electrostatic contributions from the Ppant phosphate of the holo PCP. Since no structure has been reported for HMWP2–PCP2, a homology model of HMWP2 residues 1943 to 2015 was generated in BioLuminate (74, 84) using as template a cytochrome p450–bound holo PCP crystal structure, PDB ID: 4PWV chain B (16), which shares 34% sequence identity with HMWP2–PCP2. HMWP2–PCP2 was also phosphorylated at its conserved serine residue. PCP models were prepared with neutral -CHO and -NH2 termini in BioLuminate (73, 74, 75, 76, 78) similarly to the preparation of the Cy domain models. An orthorhombic TIP3P water environment with 10 Å buffer regions was built around the HMWP2–PCP2 model using the Desmond System Builder tool in BioLuminate (74, 85, 86). The resulting system also contained 150 mM NaCl plus nine neutralizing Na^+^ ions. This system was relaxed using the default Desmond molecular dynamics (MD) relaxation protocol for the constant pressure (NPT) ensemble (see Supporting information section), then simulated at 300 K, and 1.01325 bar for 10 ns recording coordinates at 5 ps intervals (85, 86). A representative frame was selected with lowest Cα RMSD to the average model of the last 1000 frames of the trajectory (*i.e.*, a set of frames with roughly constant Cα RMSD to the input model). This model was then minimized for 100 ps using the Desmond MD energy minimization protocol. The protein model was extracted from the energy-minimized system and reprepared in the same fashion as the HMWP2–PCP1 model.

#### Cyclodehydration intermediates

BioLuminate was used to model potential phosphopantetheinylated HMWP2-Cy2 cyclodehydration intermediates of *R* and *S* chiralities at the hydroxyl-bearing carbon center of the hydroxythiazolidine ((2*R/S*,4*R*)-2,2-hydroxy-(2ʹ-(2ʺ-hydroxyphenyl)-thiazolinyl)-thiazolidinyl Ppant) (74). Note that the thiazolines of the 2-hydroxyphenyl-thiazolinyl side chains of these intermediates contain the chiral carbon corresponding to the d product of the l/d epimerization carried out by the upstream embedded E domain of HMWP2 (29). The cyclodehydration intermediate models were prepared as singly charged anions (at the phosphate) using LigPrep in BioLuminate (74, 87). These molecules will be referred to as Ppant-2HPTT(*R/S*)-OH or *R* or *S* (cyclodehydration) intermediates.

### Molecular docking

#### Protein–protein docking

The prepared HMWP2–PCP1 model was docked at the predicted opening of the upstream HMWP2-Cy2 active-site tunnel (between strands β8 and β10 of the prepared model with all water molecules removed and the modified R1812 rotamer) using default settings in the HADDOCK2.4 web server (54, 55, 56, 88) with an additional brief MD simulation in explicit solvent. Active residues providing restraints were defined as the PCP phosphoserine (HMWP2 residue SEP1439) and Cy residue S1728, near the N terminus of helix α8, where the Ppant phosphate is predicted to be in PCP_donor_–Cy complexes (based on the PCP1_donor_–C2 complex of LgrA; PDB ID: 6MFX) (51). The HMWP2–PCP2 homology model was docked similarly at the opening of the downstream active-site tunnel of HMWP2-Cy2 (between helices α1 and α10), using Cy models with each possible combination of F1513 and R1812 crystallographic rotamers and the C–PCP-mimicking R1812 rotamer. For the results presented here, active residues were Cy F1513 and R1812 and PCP F1971, SEP1977, F2001, and the Cy model contained the F1513 rotamer B, directing the phenyl toward the PCP_acceptor_ binding site, and the C–PCP-mimicking R1812 rotamer. The additional active PCP2 residues F1971 and F2001 were included to promote favorable π-stacking interactions and/or hydrophobic packing with F1513 of the Cy domain, which adopts two rotameric states in the HMWP2-Cy2 crystal structure (one flipped out toward the PCP2-binding site and one away) and is present in over 40% of selected Cy domain sequences aligned in Fig. S7. Runs were also conducted with only R1812 and SEP1977 defined as active, but these and the other rotameric combinations of F1513 and R1812 did not perform as well, in some cases returning poses that resemble PDB ID: 4ZXI or PDB ID: 6N8E but that have considerably less favorable HADDOCK scores, lower buried surface areas, and/or positive Z scores. These results are therefore not presented. In selecting docked poses, scores, energy terms, and cluster sizes output by HADDOCK were considered, and the models were visually inspected for poses that were consistent with the HMWP2 modular architecture and reasonably placed the conserved PCP serine residues (SEP1439 or SEP1977) near the upstream and downstream active-site tunnel entrances.

#### Cyclodehydration intermediates

Because reasonable HMWP2-Cy2–PCP2 didomain complex models were obtained by protein–protein docking in HADDOCK2.4, the best-ranked of them was chosen as the basis for an approximate complex of the HMWP2-Cy2 crystal structure and the docked PCP2, to provide a Ppant phosphate site. The complex was formed by superimposing the docked HMWP2-Cy2–PCP2 complex on the HMWP2-Cy2 crystal structure using Cα alignment of Cy helices α1 and α10 (the secondary structure features forming most of the downstream tunnel entrance) followed by removal of the Cy domain from docking as well as redundant and conflicting interfacial water molecules and crystallographic water molecules in the tunnel that would block substrate access. The rotameric state of R1812 of the HMWP2-Cy2 structure was altered so its guanidinium moiety is positioned similarly to guanidinium moieties of conserved arginine residues observed in C–PCP_acceptor_ complexes. The resulting complex was then preprocessed and subjected to hydrogen-bond network optimization and refinement of PCP2 and the interface waters and residues within 5 Å using the automatic method in Prime (81, 82, 83). From this model, a second was generated that contained an aspartic acid rather than aspartate at the putative catalytic dyad. Both models (*i.e.*, anionic and neutral dyads) were then subjected to hydrogen coordinate refinement followed by all-atom energy minimization in Impact with a 0.3 Å heavy atom displacement cutoff preventing the models from deviating too much from their input coordinates.

Phosphoryl groups were then removed from the conserved PCP serine residues in preparation for covalent docking using Schrödinger’s Phosphonate_Addition.cdock reaction definition file available at https://www.schrodinger.com/products/covdock (also see the Supporting information section). This reaction definition file, although designed for phosphonate reactions, is suitable for displacement of a phosphate oxygen by a serine side-chain oxygen. The covalent docking tool in BioLuminate (74) uses Glide docking (89, 90, 91) in a rigid receptor approximation and returns only poses that are compatible with the covalent linkage the user defines (92). Default covalent docking settings were used (maximum of 200 initial poses, keeping only those with Glide scores lower than +2.5, outputting the top 1000 with 30 poses per ligand reaction site, which is one site in this case). The docking grid’s inner box, within which the ligand’s center must be positioned, was 10 × 10 × 10 Å^3^ and the outer box had dimensions of 34 × 34 × 34 Å^3^. The center of the grid was defined as the centroid of tunnel-lining residues so that it was in the center of the tunnel’s diameter and positioned roughly halfway between R1812 and F1522, which lie at the ends of the docking region. Docking results were manually inspected to identify categories of hydroxythiazolidine orientation. Each category of pose for the *R* and *S* intermediates was subjected to optimization/refinement in the following scheme inspired by the refinement steps of Glide/Prime Induced Fit Docking protocols in BioLuminate (74, 81, 82, 83, 89, 90, 91, 92, 93, 94): (1) refinement of protein residues within 5 Å of the docked ligand using 10 iterations of 100 steps each with Prime’s automatic conjugate gradient/truncated Newton method, (2) refinement of the ligand and residues within 5 Å of it using 10 iterations of 100 steps each with Prime’s automatic method, (3) Monte Carlo protein side-chain optimization for residues within 5 Å of the ligand (Predict Side Chains tool running Prime in BioLuminate), and (4) refinement of the ligand and residues within 5 Å of it using 10 iterations of 100 steps each with Prime’s automatic method. These refined models were sorted by Prime energy and evaluated using a combination of that value and the docking score. As a final point of comparison between poses, the phosphate-serine linkages of top covalent docking models were cleaved by deletion of the bridging oxygen (S1977 Oγ), replacing the broken bonds with bonds to hydrogens on the separated groups to leave a phosphonate and alanine, and molecular mechanics with generalized Born and surface area minimization calculations were performed on the resulting noncovalent complexes using Prime with 1 kcal/mol·Å^2^ restraints on flexible atoms, which were defined as those of the ligand and residues within 10 Å of it (81, 82, 83).

### Low-frequency vibrational normal mode analysis

The 1.94 Å resolution structure of HMWP2-Cy2 (PDB ID: 7JTJ) was retrieved from the PDB, and the missing loop and side chains were filled using Prime in BioLuminate (74, 81, 82, 83). Using the BioLuminate Protein Preparation Wizard (72, 73, 74, 75, 76, 77, 78, 79, 80), hydrogen-bond networks were optimized, hydrogen coordinates were refined to minimize potential energy, and all atom coordinates were refined to decrease potential energy with a cutoff before heavy atom RMSD reached 0.3 Å. The PEG fragment and loosely bound surface water molecules were removed. The Desmond (85, 86) solvate pocket tool was used to add water molecules to the active-site tunnel, where the PEG fragment was removed. This model was placed in a solvent box with at least 10 Å separating the protein and the sides of the box, neutralizing with sodium ions and including 150 mM NaCl. The crystallographic sodium ion was replaced with a water molecule in this process. The default Desmond MD (85, 86) relaxation protocol was run, and the output from the last stage with solute restraints (stage 5) was used as input to a series of 100 ps MD runs in the NPT ensemble at 277 K and 1.01325 bar, gradually releasing solute restraints as follows: 10 kcal/mol·Å^2^, 5 kcal/mol·Å^2^, and then 1 kcal/mol·A^2^. These were followed with a series of 100 ps MD warming steps in 5 K increments from 280 to 300 K, each in the NPT ensemble at 1.01325 bar. An additional 5.5 ns of NPT simulation at 300 K were performed recording frames every 1 ps. A representative frame was selected from the final 2200 frames as the frame with the lowest RMSD to the average structure over this range. Surface water and ions were removed by selecting molecules more than 5 Å from the protein, then extending that selection to water or ions within 5 Å, leaving only the water most closely associated with the protein and the active-site tunnel water undisturbed. An initial energy minimization in MacroModel (Schrödinger) (95) using the Polak–Ribiere Conjugate Gradient method with a cutoff of 0.0418 kJ/mol was performed and followed by normal mode calculation in BioLuminate (74), returning 80 frames for each of the top 10 modes. This allowed for identification of loosely bound water molecules on the surface of the input model that contributed modes with negative eigenvalues. These water molecules were removed from the model used as input to the initial minimization, and the resulting model was subjected to Polak–Ribiere Conjugate Gradient energy minimization as before. Normal modes reported here were calculated for the minimized model without loosely bound surface water molecules.

### FTMap ligand fragment–binding site identification

The composite model built using the 1.94 Å and 2.35 Å resolution HMWP2-Cy2 structures was used as input to the FTMap server (60). The entire protein was defined as the search space, and default settings were used for binding site identification.

### Figure preparation

Chemical figures were generated in ChemDraw (PerkinElmer), protein figures were generated using PyMOL (Schrödinger), and electrostatics figures were produced using the APBS PyMOL plugin with charges from the OPLS3e force field available in BioLuminate and van der Waals radii (74, 78, 96). Calculations of approximate tunnel volumes were performed using the CASTp 3.0 web server available at the time of publication at http://sts.bioe.uic.edu/castp/calculation.html (97). Sequence alignments were generated using Clustal Omega (98). Representative Cy domain sequences were retrieved from UniProtKB by SANSparallel (99, 100), searching with seed Cy domain sequences from anguibactin, bacillamide, bacitracin, bleomycin, epothilone, JBIR-34/45, mycobactin, myxothiazole, pyochelin, vibriobactin, and Ybt biosynthesis. Sequence redundancy was cut at 80% in JalView (101) before alignment. WebLogo3 (102) was used to generate the sequence logo.

## Data availability

The 1.94 Å and 2.35 Å resolution crystal structures for HMWP2-Cy2 have been deposited to the PDB (http://www.rcsb.org/) and assigned the accession numbers 7JTJ and 7JUA, respectively.

All files relating to the computational studies reported here are available in the GitHub repository at https://github.com/adgnann/HMWP2-Cy2. The file README.md provides a description of the repository contents.

## Supporting information

This article contains supporting information including supplemental results and discussion as well as crystallographic data collection and refinement statistics (Table S1) and additional figures (Figs. S1–S22) (44, 45, 48, 51, 52, 57, 58, 59, 99, 100, 101, 102, 103, 104, 105). Videos of the low-frequency vibrational normal modes are also available online (Supporting movies).

## Conflict of interest

The authors declare that they have no conflicts of interest with the contents of this article.

## Supporting information

supporting information

Normal mode 1

Normal mode 2

Normal mode 3

Normal mode 4

Normal mode 5

Normal mode 6

Normal mode 7

Normal mode 8

Normal mode 9
