# Supplementary material for: High-resolution structures of a siderophore-producing cyclization domain from Yersinia pestis offer a refined proposal of substrate binding
Source: J Biol Chem. 2022 Sep 5;298(10):102454. doi: 10.1016/j.jbc.2022.102454 (PMC9547227; doi:10.1016/j.jbc.2022.102454)
Supplement: supporting information [file mmc1.pdf]

Electronic Supporting Information for:

High-resolution structures of a siderophore-producing cyclization domain from *Yersinia pestis* offer a refined proposal of substrate binding

Andrew D. Gnann[a], Yuan Xia[a,1], Jess Soule[a,2], Clara Barthélemy[a], Jayata S. Mawani[a], Sarah Nzikoba Musoke[a,3], Brian M. Castellano[b,4], Edward J. Brignole[b], Dominique P. Frueh[c], Daniel P. Dowling[a,$]

a Department of Chemistry, University of Massachusetts Boston, Boston, MA 02125
b Department of Biology, Massachusetts Institute of Technology, Cambridge, MA 02142
c Department of Biophysics and Biophysical Chemistry, Johns Hopkins University School of Medicine, Baltimore, MD 21205

1 Present address: GenScript Biotech Corporation, Piscataway, NJ, 08854
2 Present address: University of California Los Angeles, Los Angeles, CA 90095
3 Present address: Brigham and Women's Hospital, Boston, MA 02115
4 Present address: Stanford University, Stanford, CA 94305

$ To whom correspondence should be addressed: Daniel P. Dowling: Department of Chemistry, University of Massachusetts Boston, Boston, MA 02125; daniel.dowling@umb.edu; Tel. (617) 287-5853; Fax. (617) 287-6030

Files for computational work are available at https://github.com/adgnann/HMWP2-Cy2

Contents

Supplemental Results Page 1

Supporting Information Table S1 Page 2

Supporting Information Figures Page 3

Supplemental Results

Additional Cyclodehydration Intermediate Docking Observations

Relationship between intermediate poses and crystallographic water sites

As was noted in the main-text docking results, the *R* cyclodehydration intermediate poses with their leaving group oxygen atoms directed toward α4 are compatible with a crystallographic water in this area, and the leaving group oxygen resembles another crystallographic water. *S* cyclodehydration intermediate poses with their leaving group oxygen atoms toward α4 are positioned differently so that both crystallographic water sites could be accommodated in addition to the leaving group. How these water molecules behave during catalysis will be interesting for further study.

Description of less probable intermediate docking categories

Although deemed less probable based on docking metrics, conservation and positioning of substrate reactive groups in relation to active site residues, other intermediate pose categories are discussed here. The two additional distinct conformational categories of *S* cyclodehydration intermediate poses (hydroxyl-toward-N-terminal-sheet and hydroxyl-toward-dyad) are initially intriguing because they also show favorable interactions with the Ppant arm and place reactive groups in the vicinity of conserved active site features (Figs. S14-15). However, it should be considered that the poses placing the hydroxyl toward the N-terminal β sheet would imply a substantial conformational change rotating the leaving group oxygen from its envisioned position during condensation (at the N-terminus of α4) through a large angle with concomitant rearrangement of the bulky 2HPT side chain and presumably of the Ppant arm as well. Similarly, the poses placing the leaving group oxygen toward the dyad also imply a large conformational change, and it is unclear where the cysteine thiol nucleophile would be sequestered during condensation if the cysteine amine is the initial nucleophile. With respect to the hydroxyl-toward-dyad category, one may envision how an initial thiol condensation with the amine nitrogen sequestered near T1856 could occur. One unique *S* intermediate pose was notable for its reference-like dimethyl positioning, rearrangement of Q1858 to form an interaction with the Ppant hydroxyl, and favorable interactions between N1751 and T1856 and the Ppant amide nearest the thioester and the thioester carbonyl, respectively. The 2HPT side chain is also deeper into the hydrophobic pocket in the side chain-binding region formed largely by F1522, M1629, F1840 and Y1866. This pose, however, also necessitates a sizable rotation of the hydroxythiazolidine and 2HPT side chain from the envisioned post-condensation state to the state it represents.

The other *R* intermediate categories may face the same complications as those of the *S* intermediates with leaving group oxygen atoms near the dyad or pointing toward the N-terminal subdomain β sheet. It appears that the *R* intermediate category of poses with the oxygen leaving group directed toward the dyad would, however, be obtainable from the envisioned condensation state with the cysteine amine as the initial nucleophile, suggesting the catalytic dyad could act as an acid protonating the leaving group in the cyclodehydration reaction. Poses of this category also direct the Ppant dimethyl moiety toward I1808, reminiscent of the orientation AB3403 (PDB ID 4ZXI) and FscG (PDB ID 7KW0) (Drake et al., 2016; Izoré et al., 2021).

Potential interactions in the donor side-chain binding region

Poses of both intermediate diastereomers place the 2HPT side chain in the vicinity of the nonconserved residues F1522 and M1629 in the side chain-binding region. For the *R* intermediate, this arrangement of its side chain and F1522 could permit M1629 to pack against their phenyl rings, which could offer a specific aromatic-methionine-aromatic interaction that would help select the cognate substrate. The S poses are positioned so that it is nearly possible for staggered parallel stacking between the 2HPT phenyl and F1522, and this positioning is compatible with interactions between the 2HPT hydroxyl and N1621, although it should be noted that this residue is a phenylalanine in the second Cy domain of pyochelin biosynthesis. In the *R* poses, the N1621 carboxamide is often stacked on the thiazoline ring of the 2HPT side chain, sometimes forming a polar contact with S1854. The positioning of N1621 in many of the *S* poses places it in direct contact with the leaving group oxygen, a situation that seems unlikely since this residue is not conserved and its position in the sequence is often occupied by a hydrophobic residue.

References

Drake, E.J., Miller, B.R., Shi, C., Tarrasch, J.T., Sundlov, J.A., Allen, C.L., Skiniotis, G., Aldrich, C.C., and Gulick, A.M. (2016). Structures of two distinct conformations of holo-non-ribosomal peptide synthetases. *Nature* **529**, 235-238.

Izoré, T., Candace Ho, Y.T., Kaczmarski, J.A., Gavriilidou, A., Chow, K.H., Steer, D.L., Goode, R.J.A., Schittenhelm, R.B., Tailhades, J., Tosin, M., *et al.* (2021). Structures of a non-ribosomal peptide synthetase condensation domain suggest the basis of substrate selectivity. *Nat. Commun*. **12**, 2511.

Supporting Information Table

Table S1 – Data collection and refinement statistics.

	HMWP2-Cy2 at 1.94 Å resolution	HMWP2-Cy2 at 2.35 Å resolution
Data Collection		
**Detector**	Saturn 944+	ADSC Quantum 315.1
**Space group**	$P4_12_12$	$P4_12_12$
**a,b,c**	89.29, 89.29, 140.07	89.32, 89.32, 140.04
**Resolution range (Å)***	30.8 - 1.94 (1.99 – 1.94)	50.0 - 2.35 (2.43 – 2.35)
**Completeness**	98.9 (96.1)	99.3 (99.3)
**Redundancy**	16.449 (9.418)	11.8 (12.1)
**Unique Reflections**	41892 (2957)	24196 (2349)
**I/σI**	28.25 (3.41)	19.2 (2.94)
R_{sym}#	0.077 (0.735)	0.142 (0.853)
$R_{pim}$$	0.019 (0.236)	0.043 (0.252)
**CC1/2 &**	1.00 (0.848)	0.994 (0.851)
Refinement		
**Resolution range (Å)**	30.8 – 1.94	23.33 – 2.35
R_{free} ^	0.2167	0.2117
R_{work} ^	0.1799	0.1717
**Reflections/test set**	41875/2074	24158/1200
**Number of atoms**		
**Protein**	3836	3477
**Water**	355	227
**Sodium and PEG**	9	9
**B-factors (Å^2)**	32.80	38.33
**Protein**	32.17	38.23
**Water**	38.29	39.31
**Sodium**	27.30	35.80
**PEG**	60.02	57.09
**RMS Deviations**		
**Bonds (Å)**	0.008	0.003
**Angles (°)**	0.89	0.56
**Dihedrals (°)**	17.75	17.20
**Ramachandran Analysis†**		
**Favored (%)**	98.81	99.05
**Allowed (%)**	1.19	0.47
**Outliers (%)**	0.0	0.47

† Ramachandran analysis calculated using MolProbity (Williams et al., 2018).
* Highest resolution shell is shown in parentheses
\# $R_{sym/merge} = \Sigma_{hkl}\Sigma_i|I_i(hkl) - \langle I(hkl)\rangle|/\Sigma_{hkl}\Sigma_i I_i(hkl)$.
$ $R_{pim} = \Sigma_{hkl}[1/(N-1)]^{1/2}\Sigma_i|I_i(hkl) - \langle I(hkl)\rangle|/\Sigma_{hkl}\Sigma_i I_i(hkl)$ where $I_i(hkl)$, $\langle I(hkl)\rangle$ and N represent the intensity measurement, the mean intensity, and the redundancy for reflection hkl, respectively.
& $CC^* = [2CC_{1/2}/(1+CC_{1/2})]^{1/2}$ where $CC_{1/2}$ is the correlation between two random halves of the datasets, each containing half of the measured intensities for each unique reflection and CC* is an approximation of the correlation coefficient for a noise-free dataset.
^ $R_{work} = \Sigma|F_{obs}(hkl) - F_{calc}(hkl)|/\Sigma|F_{obs}(hkl)|$, where $F_{obs}(hkl)$ and $F_{calc}(hkl)$ are the observed and calculated structure factor amplitudes of ~95% of the reflections used for refinement. R_{free} was calculated from the ~5% of total reflections that were omitted from the refinement.

Supporting Information Figures

Figure	Page
Figure S1 - (methyl)ox-/thiazol((id)ine) natural products.	4
Figure S2 - A mechanistic proposal for reactions in the Cy domain.	5
Figure S3 – Secondary structure assignment in HMWP2-Cy2.	6
Figure S4 – Size exclusion chromatography verifying the monomeric state of HMWP2-Cy2.	7
Figure S5 – Tunnel representations and solvent-accessible measurements from the CASTp 3.0 server for three Cy domain crystal structures.	8
Figure S6 – Crystallographic sodium site in the active site tunnel at the interface of strands β1 (N-terminal subdomain) and β11 (C-terminal subdomain).	9
Figure S7 – Annotated sequence logo representing 1040 Cy domain sequences.	10
Figure S8 – Alignment of the DXXXXD(XXS) motif of sequences used in SANSparallel to search Uniprot KB.	11
Figure S9 – Required displacements at the upstream tunnel entrance of HMWP2-Cy2 (cyan) to achieve an open state.	12
Figure S10 – Variation in loops of the downstream tunnels of Cy domains and representative C-PCP$_{acceptor}$ models.	13
Figure S11 – Comparison of downstream tunnel entrances in Cy domains.	14
Figure S12 – Cyclodehydration intermediate models used in covalent docking experiments.	15
Figure S13 – Hydroxyl-toward-α4 Ppant-2HPTT(*S*)-OH poses from covalent docking.	16
Figure S14 – Hydroxyl-toward-N-terminal-sheet Ppant-2HPTT(*S*)-OH poses from covalent docking.	17
Figure S15 – Ppant-2HPTT(*S*)-OH covalent docking poses in hydroxyl-toward-dyad or deeply placed hydroxyl-toward-N-terminal-sheet orientations.	18
Figure S16 – Hydroxyl-toward-α4 Ppant-2HPTT(*R*)-OH poses from covalent docking.	19
Figure S17 – Hydroxyl-toward-N-terminal-sheet Ppant-2HPTT(*R*)-OH poses from covalent docking.	20
Figure S18 – Hydroxyl-toward-dyad Ppant-2HPTT(*R*)-OH poses from covalent docking.	21
Figure S19 – Geometric rationale for a proposed pre-condensation state shared by C and Cy domains.	22
Figure S20 – Comparison of the top cyclodehydration intermediate pose from HMWP2-Cy2 and the product-bound state reported for PchE-Cy.	23
Figure S21 – The high-amplitude HMWP2-Cy2 low-frequency normal mode number 7.	24
Figure S22 – The HMWP2-Cy2 active site binds small organic molecules in FTmap docking.	25

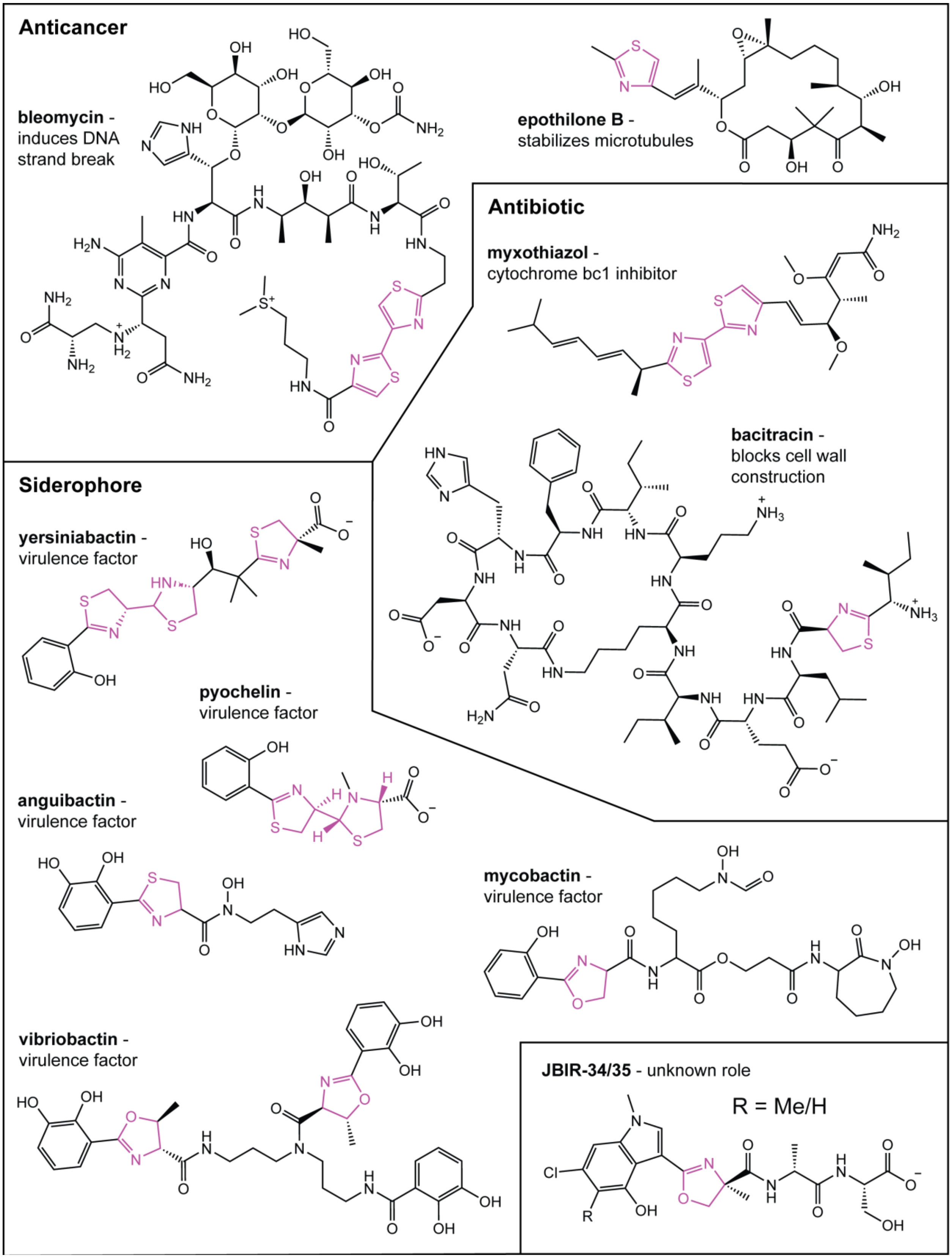

Figure S1 - (methyl)ox-/thiazol((id)ine) natural products.

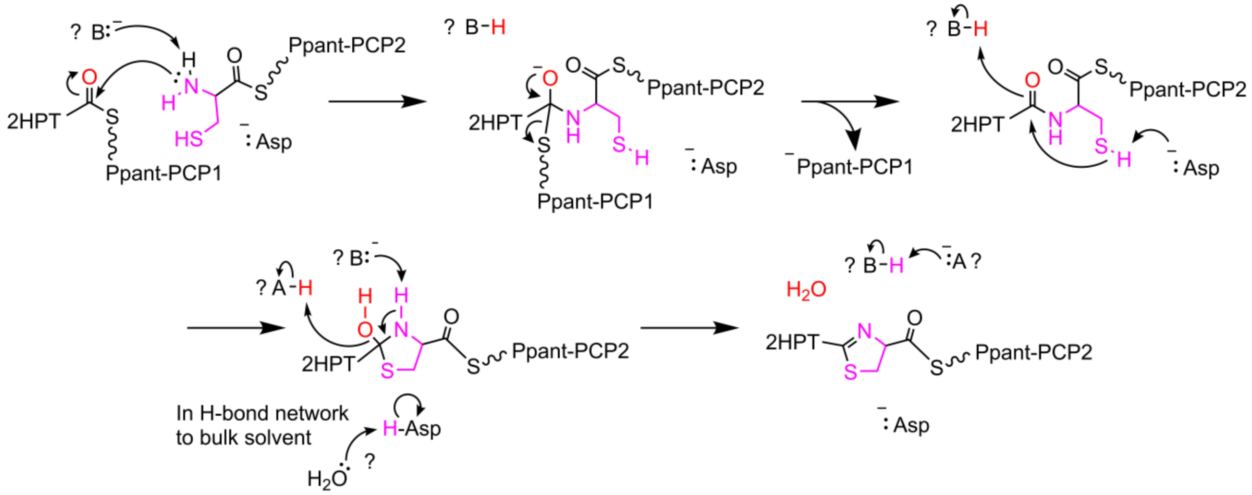

Figure S2 - A mechanistic proposal for reactions in the Cy domain. The oxygen incorporated in the water leaving group is colored red. The acceptor cysteine side chain and amine N-H incorporated into the condensation product are colored magenta. Some outstanding questions regarding catalysis by Cy domains involve the nature of proton transfer steps. Unknown species invoked to participate in these steps are indicated by question marks. The possibility of the first step involving a cysteine thiolate nucleophile instead of the amine nucleophile cannot be ruled out at this time, but it is not shown here.

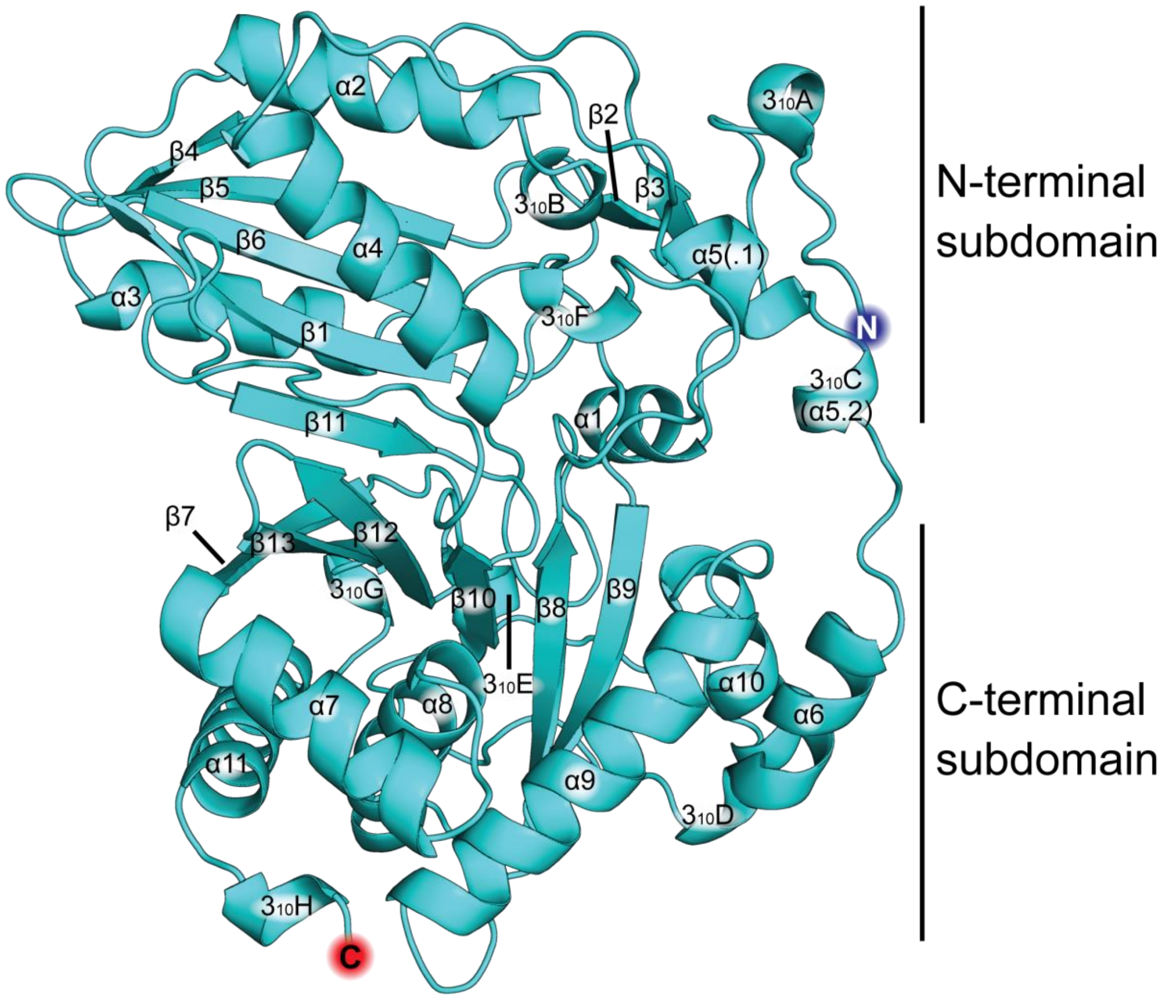

Figure S3 – Secondary structure assignment in HMWP2-Cy2. The model depicted here primarily contains coordinates from the higher resolution structure with the exception that residues missing from that structure in the region between 3_{10}C and α6 (HMWP2 1664-1665 and 1667-1668) are modeled using coordinates from the lower resolution structure. Prime in BioLuminate was used to model P1666, the final missing residue in that region. The N- and C-terminal chloramphenicol acetyltransferase-like subdomains are labeled, and the N- and C termini are marked with blue and red spheres, respectively. 3_{10} helices identified by STRIDE (Frishman and Argos, 1995) are displayed in helix cartoon representations, and they are defined to include all residues bearing a hydrogen bond donor/acceptor interacting within the 3_{10} helix as identified by manual inspection, which extends the STRIDE prediction by 1 residue in some cases (these definitions are reflected in Fig. S7).

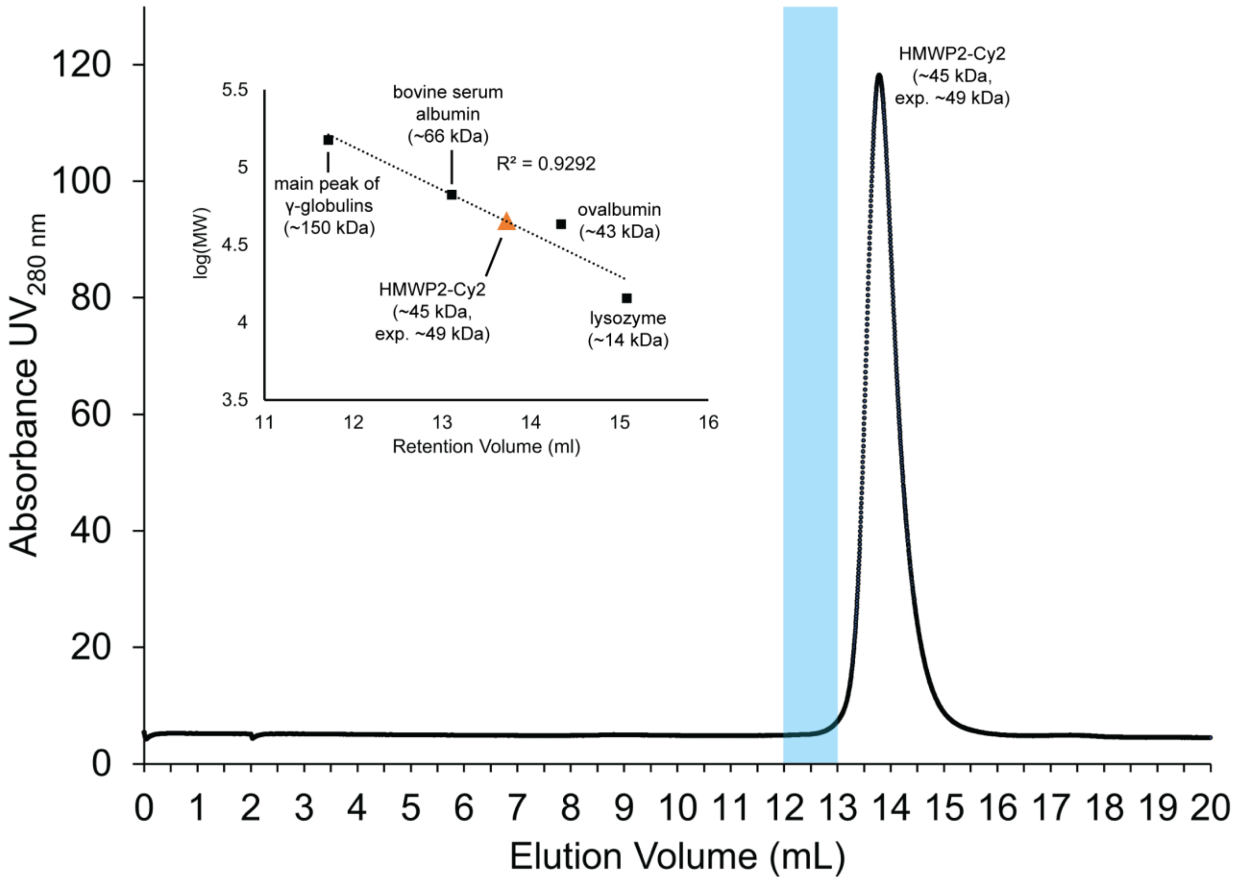

Figure S4 – Size exclusion chromatography verifying the monomeric state of HMWP2-Cy2. The main plot of absorbance at 280 nm versus elution volume shows a single, homogenous peak for HMWP2-Cy2 corresponding to a molecular weight just lower than expected (~45 kDa versus the expected ~49 kDa). The blue stripe centered on 12.5 mL indicates the volume around which a dimer of HMWP2-Cy2 would be expected to elute. No feature is observed in this region. The inset panel shows the regression curve fitting the logarithm of molecular weight versus retention volume for four protein standards, as well as an orange triangle marker for HMWP2-Cy2 and the R^2 value of the fit, 0.9292.

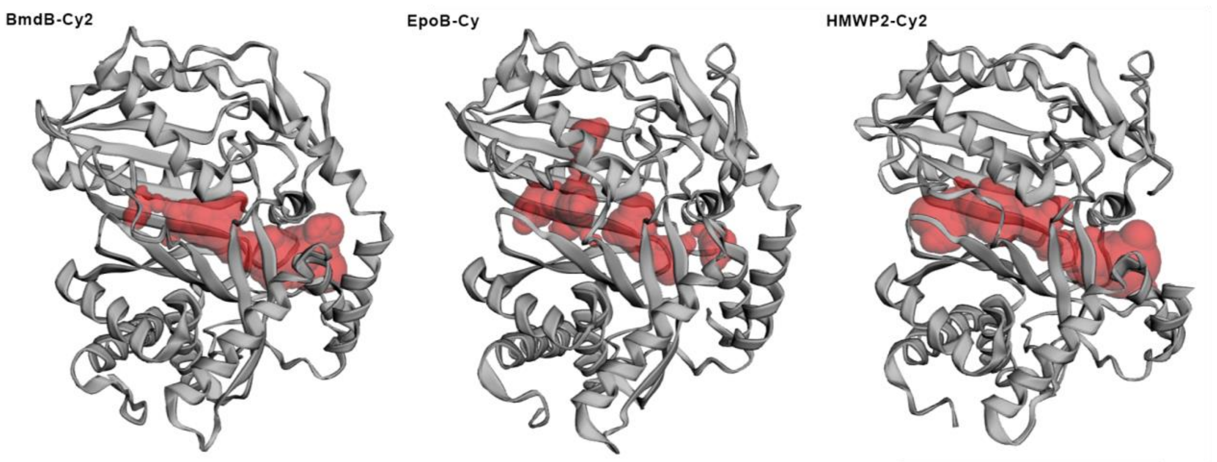

Pocket ID	SA Surface Area (Å²)	SA Volume (Å³)
1	625.675	382.242

Pocket ID	SA Surface Area (Å²)	SA Volume (Å³)
2	316.777	105.019
4	179.873	73.007
9	60.498	20.570

Pocket ID	SA Surface Area (Å²)	SA Volume (Å³)
1	774.503	543.174

Figure S5 – Tunnel representations and solvent-accessible measurements from the CASTp 3.0 server for three Cy domain crystal structures. These panels are taken from output in the graphical user interface of CASTp 3.0. Note that the tunnel in the EpoB-Cy crystal is formed by a composite of three tunnel definitions, indicating that the tunnel is discontinuous to a probe of water radius (1.4 Å) in this model. The sum of the EpoB-Cy volumes is 198.6 Å^2. (BmdB-Cy2 PDB ID 5T3E (Bloudoff et al. 2017), EpoB-Cy PDB ID 5T7Z (Dowling et al. 2016), HMWP2-Cy2 PDB ID 7JTJ)

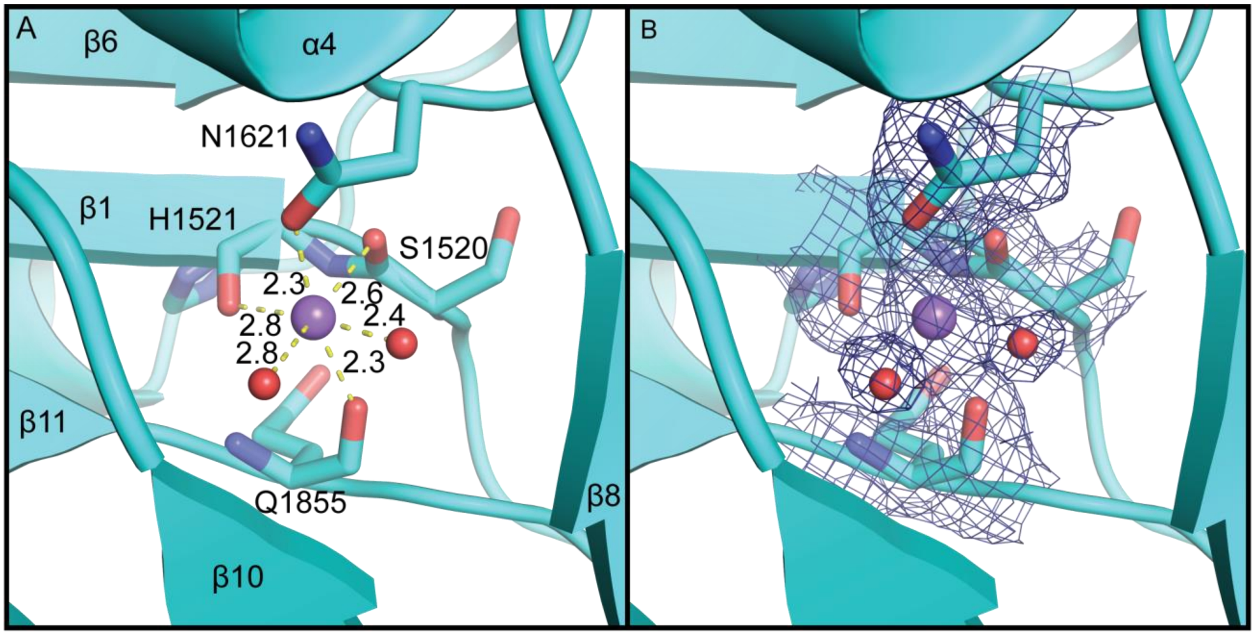

Figure S6 – Crystallographic sodium site in the active site tunnel at the interface of strands β1 (N-terminal subdomain) and β11 (C-terminal subdomain). A, Distances between oxygen ligands and the sodium ion are labeled in Å (yellow dashed lines). **B**, composite omit $2mF_o\text{-}DF_c$ electron density map contoured at 1 σ. Mesh is displayed within 2 Å of S1520, H1521 and Q1855 backbone atoms, N1621 side chain atoms and the two displayed water oxygen atoms.

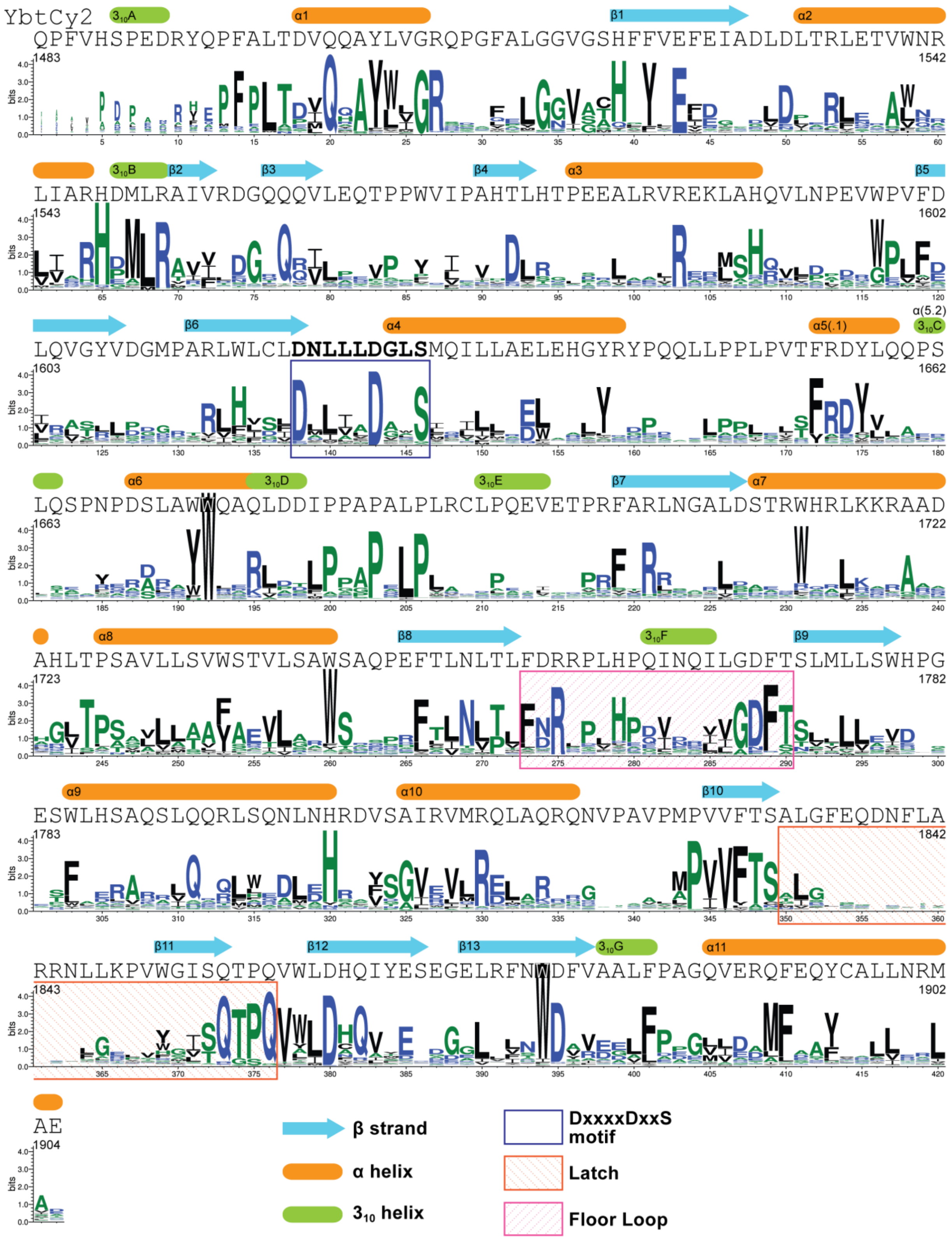

Figure S7 – Annotated sequence logo representing 1040 Cy domain sequences. The sequence of HMWP2-Cy2 is aligned above the logo. Secondary structure annotations correspond to features identified by STRIDE (Frishman and Argos, 1995) in the HMWP2-Cy2 model, and 3_{10} helix definitions match those in Fig. S3. Seed sequences used to search UniProtKB with SANSparallel (Koskinen and Holm, 2012; Somervuo and Holm, 2015) were from anguibactin, bacillamide, bacitracin, bleomycin, epothilone, JBIR-34/35, mycobactin, myxothiazole, pyochelin, vibriobactin, and yersiniabactin biosynthesis. JalView (Waterhouse et al, 2009) was used to perform an 80% redundancy cut of sequences longer than 400 amino acids, providing 1040 sequences for alignment by Clustal Omega (Sievers et al., 2011). The sequence logo in this figure was generated using WebLogo3 (Crooks et al., 2004).

HMWP2-Cy2	DNLLLDGLS	
BlmIV-Cy1	DALICDAHS	42.72
BlmIV-Cy2	DLLIADAHS	41.29
MtaC-Cy1	DAITADASA	39.68
HMWP1-Cy3	DLLQFDVQS	39.23
MtaD-Cy2	DLLTADAFS	39.04
PchF-Cy2	DFTLVDYAS	38.86
PchE-Cy1	DLLAADVES	38.28
BacA1-Cy1	DPLICDDSS	37.83
HMWP2-Cy1	DLLIMDASS	36.87
FmoA3-Cy	DLQLMDASS	35.78
BmdB-Cy2	DALLMDGAS	35.20
EpoB-Cy	DLINVDLGS	35.03
MbtB-Cy1	DMQAADAMS	32.57
VibF-Cy1*	DMIACDAQS	27.44
AngN-Cy1	DMIAIDPDS	25.88
VibF-Cy2†	DALIVDGRT	25.81
AngN-Cy2	DALILDARS	21.99

Figure S8 – Alignment of the DXXXXD(XXS) motif of sequences used in SANSparallel to search UniProtKB. The sequences are sorted by identity to HMWP2-Cy2 (percent ID is in the right column). Yellow squares indicate Cy domains using cysteine, red squares indicate Cy domains using hydroxyl-bearing acceptors. Blue boxes mark the conserved positions of the motif. The black brackets in the bottom left indicate pairs of tandem Cy domains.

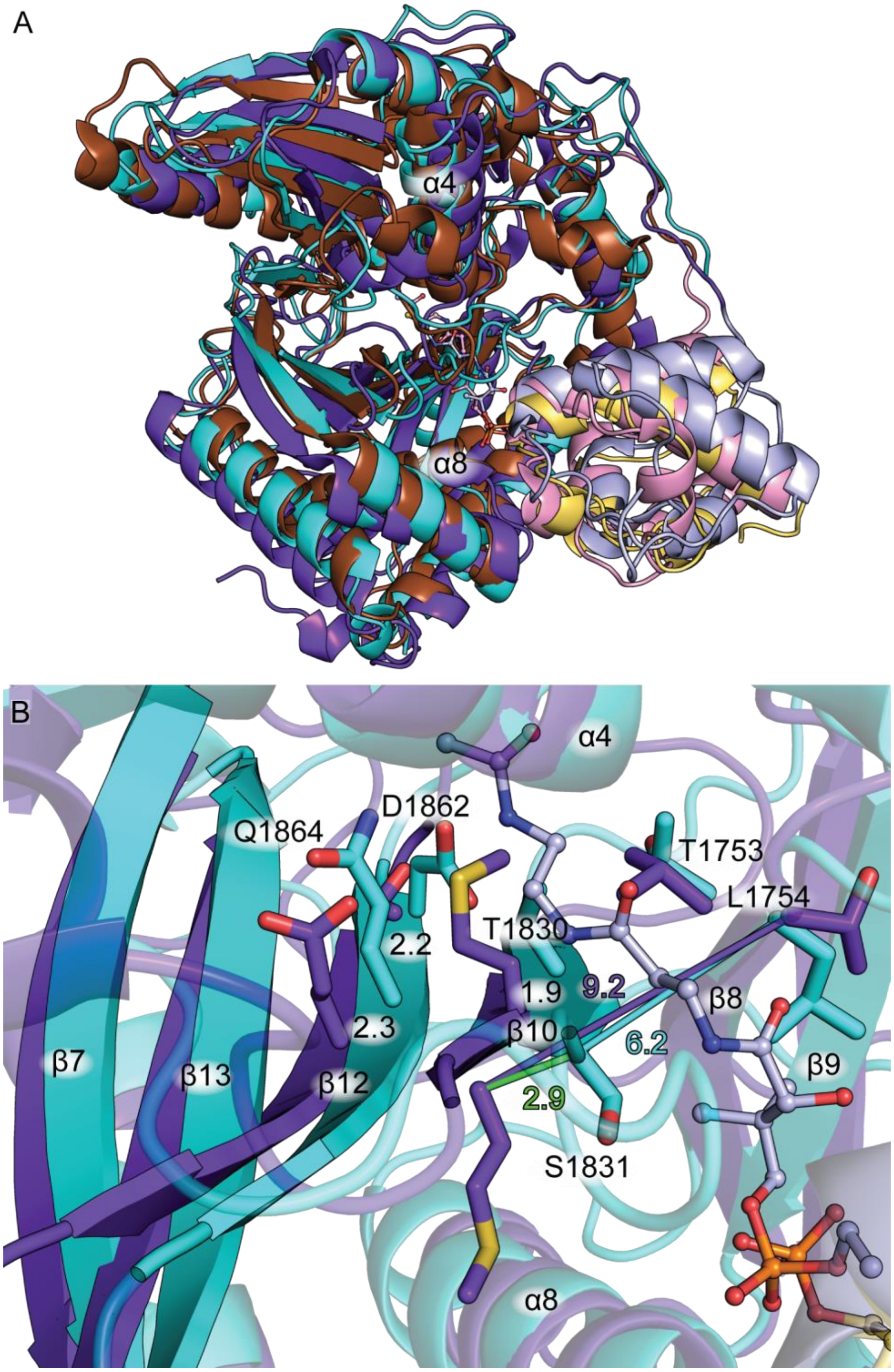

Figure S9 – Required displacements at the upstream tunnel entrance of HMWP2-Cy2 (cyan) to achieve an open state. A, A superimposition of the protein-protein docking model of HMWP2-PCP1-Cy2 (PCP yellow, Cy cyan; see also Fig. 3A-D) with LgrA-C (C purple, PCP light blue, Ppant analog light blue ball and stick, PDB ID: 6MFX, Reimer et al. 2019) and PchE-Cy (Cy brown, PCP pink, Ppant pink ball and stick, PDB ID: 7EN1, Wang et al. 2022). **B**, The gap between strand β8 leading into the floor loop and strand β10 leading into the latch would expand by nearly 3 Å (from ~6.2 Å in HMWP2-Cy2 to 9.2 Å in LgrA-C) to accommodate binding of the donor pantetheine. Approximately 2 Å also separate the Cα atoms of the Cy catalytic dyad from the Cα atoms of their counterparts in LgrA-C, reflecting that the entire lobe of the C-terminal subdomain sheet containing β-strands 7, 13, 12, and 10 is rotated.

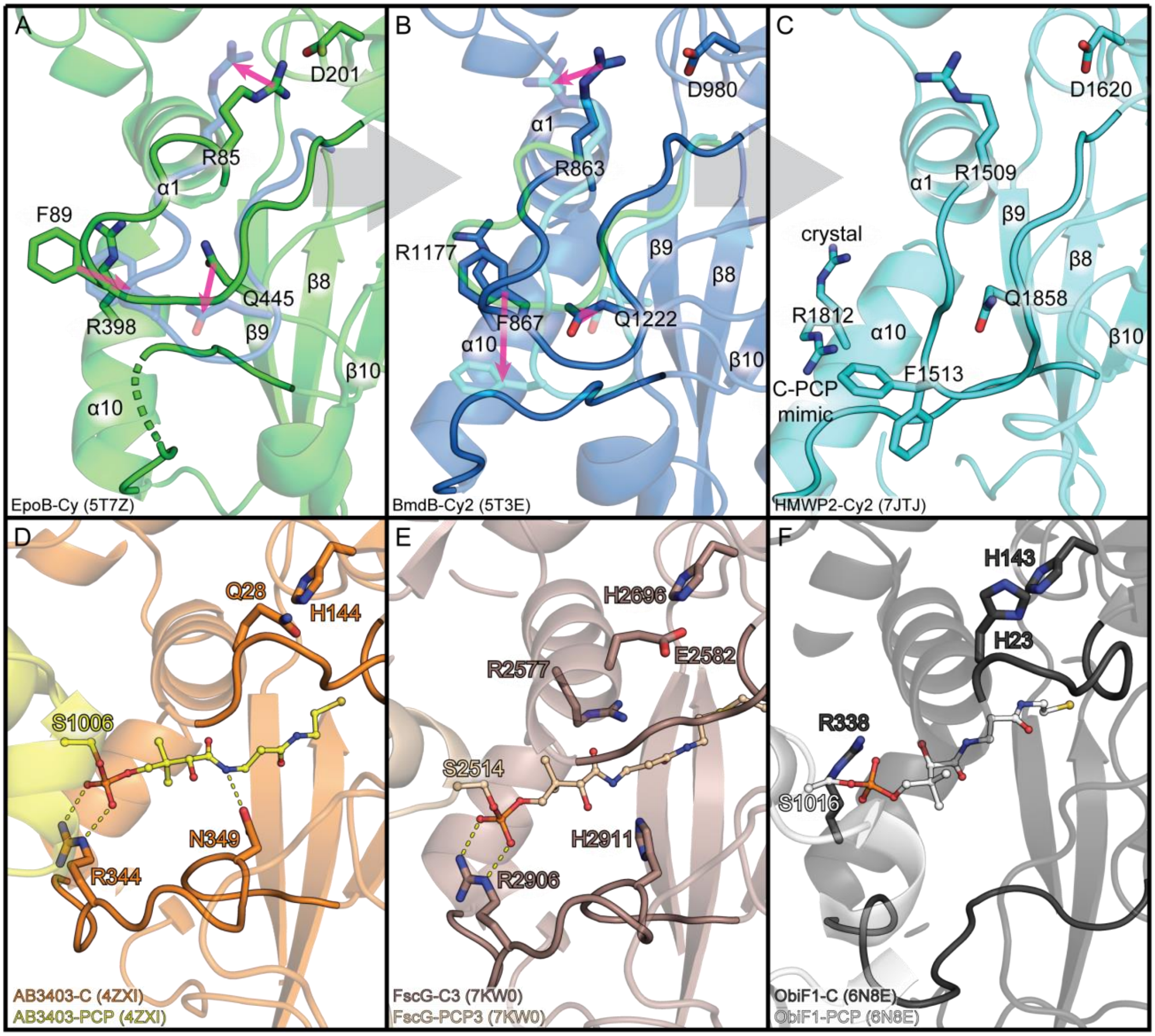

Figure S10 – Variation in loops of the downstream tunnels of Cy domains and representative C-PCP$_{acceptor}$ models. The system/name and PDB ID of each model is listed in the bottom left corner of each panel. **A**, EpoB-Cy is displayed in green. The blue, transparent cartoon/stick overlay shows the position of loop 1 in BmdB-Cy2 for direct comparison. Pink arrows highlight the movements of loop 1 residues displayed in sticks. **B**, BmdB-Cy2 is displayed in blue. The cyan and green transparent cartoon/stick overlays show the positions of loop 1 in EpoB-Cy and HMWP2-Cy2 for direct comparison, and pink arrows highlight movements of loop 1 residues between BmdB-Cy2 coordinates and HMWP2-Cy2 coordinates. **C**, The downstream tunnel entrance state in HMWP2-Cy is shown. Both crystallographic F1513 rotamers are displayed, and the alternate R1812 rotamer used in docking as well as the crystallographic R1812 rotamer are shown. **D**, The C-PCP$_{acceptor}$ model from AB3403 is displayed, showing the R344-S1006 (phosphate) interaction that inspired testing the alternate HMWP2-Cy2 R1812 rotamer, and a single hydrogen bond between N349 and the pantetheine (yellow ball and stick representation) (Drake et al. 2016). The visible secondary structure shares the same numbering as the Cy domains in panels A-C. **E and F**, The same view as panel D is shown, but for FscG (Izoré et al. 2021) and ObiF1 (Kreitler et al. 2019). Whereas FscG-C3 offers a potential polar contact to pantetheine at H2911, ObiF1 shows no polar contact to pantetheine in this position. Additionally, ObiF1 demonstrates a different downstream tunnel entrance arginine configuration that resembles the Cy domains (R338) and the pantetheine is positioned differently so that the thiol is not as deep into the active site region and the dimethyl moiety is rotated around the axis of the pantetheine by approximately 180° relative to AB3403 and FscG models.

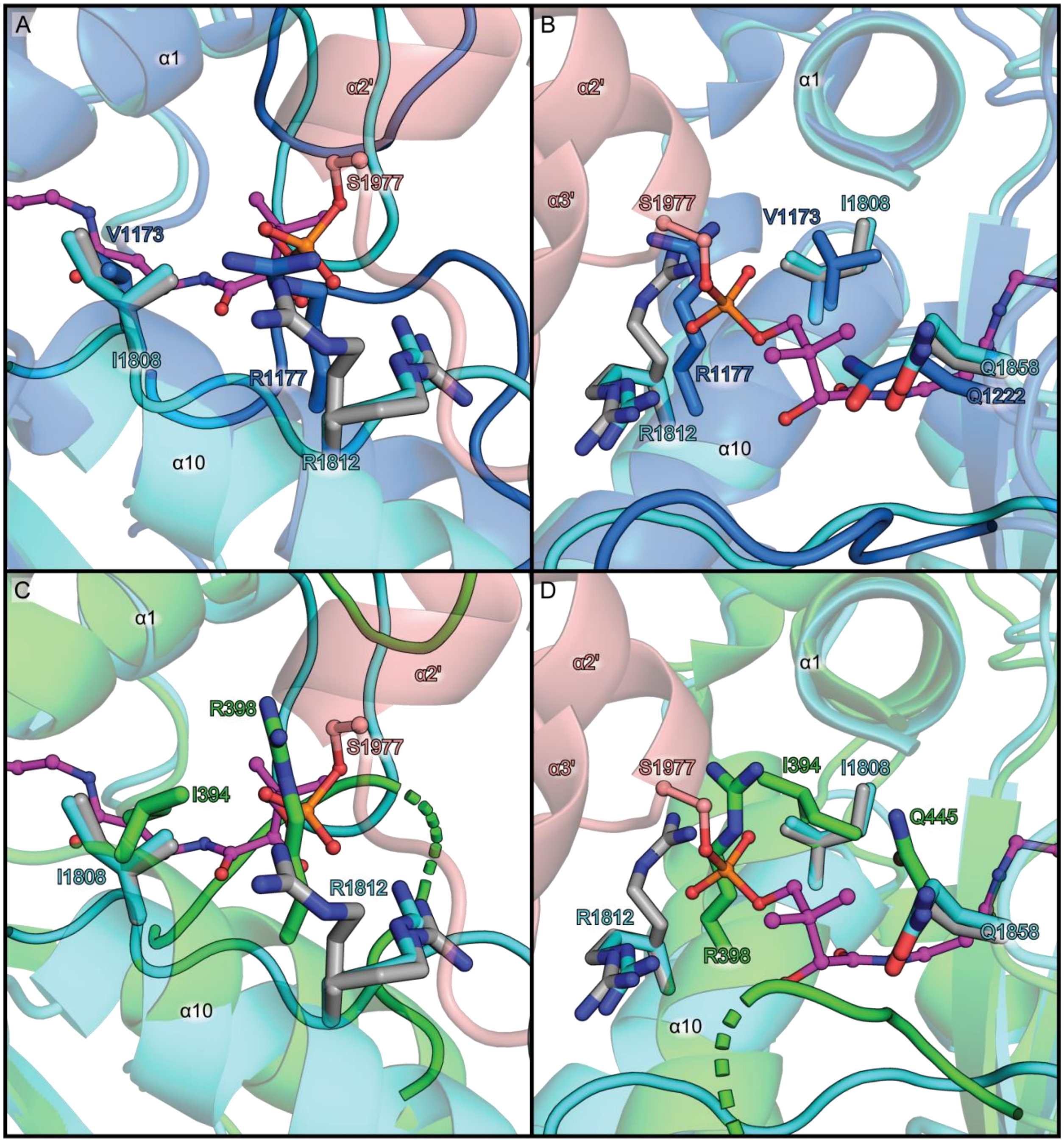

Figure S11 – Comparison of downstream tunnel entrances in Cy domains. A and B, BmdB-Cy2 (blue) is superimposed on the docked HMWP2-Cy2/HMWP2-PCP model (cyan/salmon) with the top *R* cyclodehydration intermediate pose (magenta ball and stick, described further in the results section on cyclodehydration intermediate docking and Figs. 4 and S17-23). Gray sticks are HMWP2-Cy2 crystal structure coordinates or the docking input model (R1812 alternate conformation). Non-transparent cartoon loops are the loops following α1 and α10. Panel B is approximately related to panel A by a 140° rotation around the Y axis. Note that both the crystallographic and C-PCP$_{acceptor}$-based HMWP2-R1812 rotamers are displayed in panel B. Also, the conserved glutamines (HMWP2-Q1858 and BmdB-Q1222) are in different rotameric states. **C and D**, The same views are shown as in panels A and B, but for comparison of HMWP2-Cy2 and EpoB-Cy (green). In HMWP2-Cy2, EpoB-Cy and BmdB-Cy2, a small hydrophobic residue (I/V) is found near the N-terminus of helix α10 in the vicinity of the pantetheine dimethyl groups of the C-PCP models from AB3403 and FscG (PDB IDs 4ZXI or 7KW0, respectively), not ObiF1 (PDB ID 6N8E).

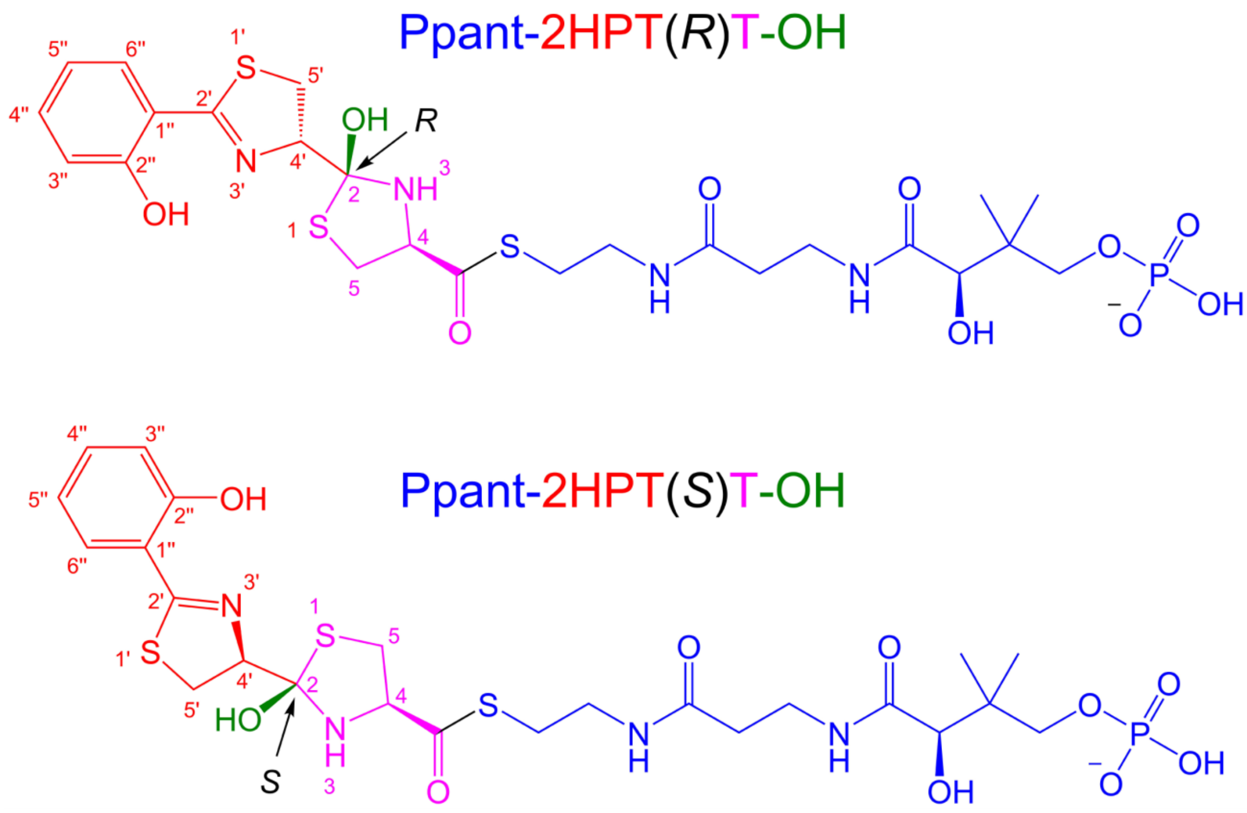

Figure S12 – Cyclodehydration intermediate models used in covalent docking experiments. Structures of both chiralities of cyclodehydration intermediates tested here are color coded by moiety. The 2HPT side chain is in red, the hydroxyl (water leaving group) is in green, the thiazolidine and carbonyl derived from cysteine in HMWP2-Cy2 is in magenta, and the phosphopantetheine is in blue. The structures differ only by their configuration at the 2 position of the reactive hydroxythiazolidine moiety. The structures are displayed in such a way that the orientation of the side chain roughly resembles its orientation in the top covalent docking results for each intermediate (the hydroxyl-toward-α4 classes of pose). Ring numbering uses primes (') to differentiate between rings.

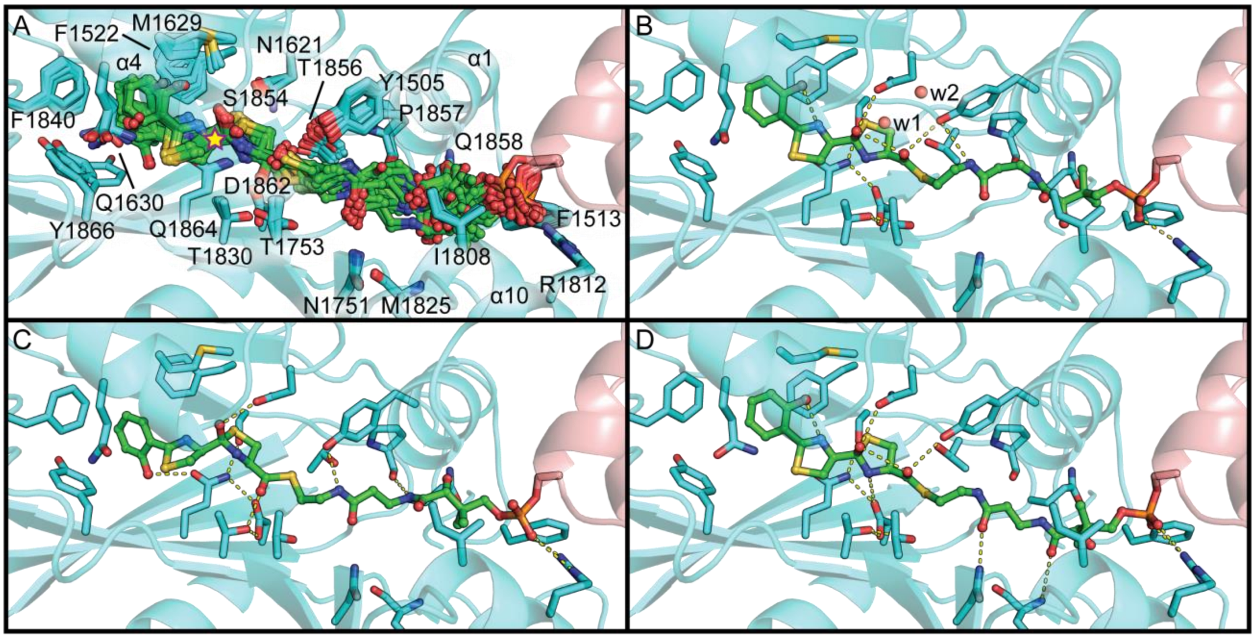

#	Panel	Dyad State	Glide Docking Score	Prime Energy kcal/mol	Prime MM-GBSA: dG Bind kcal/mol	Complex Energy kcal/mol	Receptor Energy kcal/mol	Receptor Strain Energy kcal/mol	Ligand Energy kcal/mol	Ligand Strain Energy kcal/mol
1	B	neutral	-8.3	-20949	-62	-21090	-20918	3.5	-109	19
2	C	neutral	-11.2	-20946	-75	-21084	-20913	7.1	-95.4	11
3	D	neutral	-10.8	-20943	-63	-21085	-20916	5.9	-106	17

Figure S13 – Hydroxyl-toward-α4 Ppant-2HPTT(*S*)-OH poses from covalent docking. HMWP2-Cy2 is in cyan, and Ppant-2HPTT-OH is in green. **A**, A superimposition of the class of poses in which the leaving group oxygen is directed toward the N-terminus of helix α4 is shown. A yellow star marks the leaving group oxygen. This class consistently positions the pantetheine so that Y1505 can interact with the thioester or the adjacent pantetheine amide. In these poses, Y1505 is too far from the reactive hydroxythiazolidine ring to form direct interactions with it. Both orientations of 2HPT are observed. **B-D**, Representative top poses are displayed in the same order as in the table at the bottom of the figure (sorted by Prime Energy of the refined covalent docking complex). The crystallographic water sites near the N-terminus of helix α4 are displayed in panel B for comparison against the top *R* hydroxyl-toward-α4 pose. The pantetheine dimethyl group is found in orientations resembling either AB3403/FscG (PDB IDs 4ZXI or 7KW0, respectively) or ObiF1 (PDB ID 6N8E). The pose in panel C places the hydroxyl to the side of the N-terminus of helix α4. The table displays docking scores and energy properties for each pose in panels B-D. Table coloration uses the same scale in Figs. S13-18 and serves to highlight more favorable poses. Darker green corresponds to favorable energy properties. Darker red corresponds to less favorable strain energy terms.

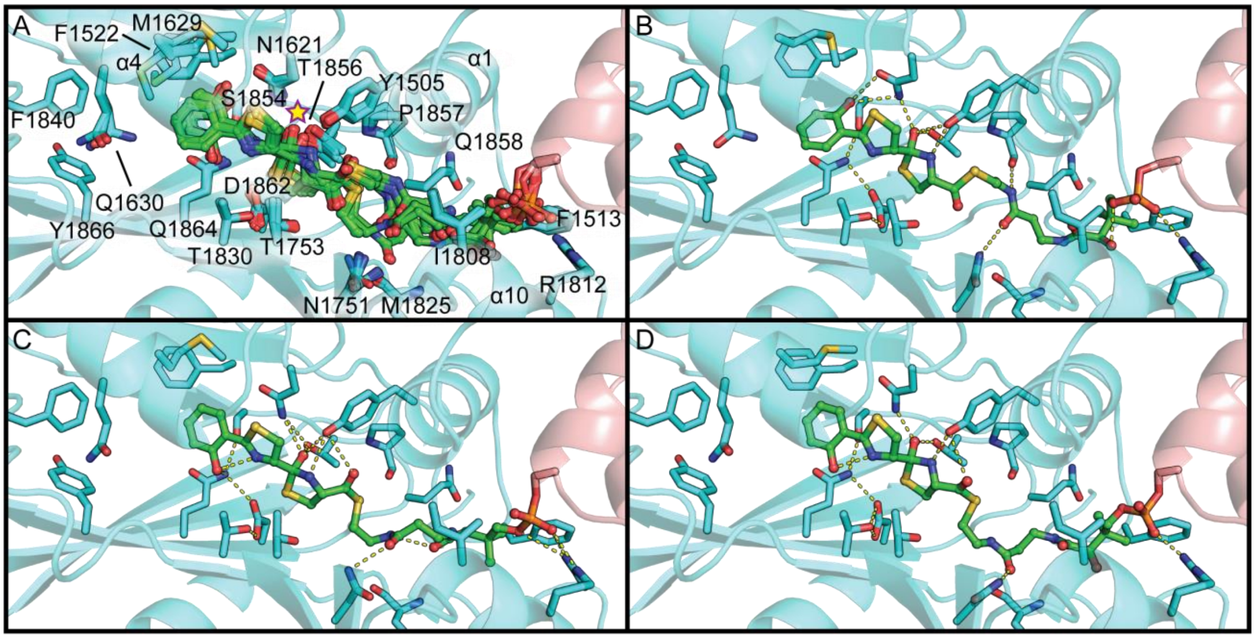

					Prime MM-GBSA					
#	Panel	Dyad State	Glide Docking Score	Prime Energy kcal/mol	dG Bind kcal/mol	Complex Energy kcal/mol	Receptor Energy kcal/mol	Receptor Strain Energy kcal/mol	Ligand Energy kcal/mol	Ligand Strain Energy kcal/mol
4	B	neutral	-10.2	-20932	-76	-21072	-20900	11	-96.5	12
5	C	neutral	-10.6	-20924	-60	-21072	-20914	11	-101	20
6	D	negative	-8.9	-20917	-68	-21066	-20896	12	-83.7	26

Figure S14 – Hydroxyl-toward-N-terminal-sheet Ppant-2HPTT(*S*)-OH poses from covalent docking. HMWP2-Cy2 is in cyan, and Ppant-2HPTT-OH is in green. **A**, A superimposition of the class of poses in which the leaving group oxygen is directed toward the N-terminal subdomain β sheet is shown. A yellow star marks the leaving group oxygen. This class positions the pantetheine thioester so that Y1505 can interact with its carbonyl oxygen, but Y1505 also forms interactions with the hydroxythiazolidine ring nitrogen and the leaving group oxygen in some poses. Both orientations of 2HPT are observed. **B-D**, Representative top poses are displayed in the same order as in the table at the bottom of the figure (sorted by Prime Energy of the refined covalent docking complex). All these poses position the pantetheine dimethyl similarly to ObiF1 (PDB ID 6N8E). These poses all necessitate a flip of the leaving group oxygen from toward the N-terminus of helix α4 (where it would be expected to reside following the condensation reaction) to toward the N-terminal sheet, which would likely also necessitate substantial motion of the 2HPT side chain. The table displays docking scores and energy properties for each pose in panels B-D. Table coloration uses the same scale in Figs. S13-18 and serves to highlight more favorable poses. Darker green corresponds to favorable energy properties. Darker red corresponds to less favorable strain energy terms.

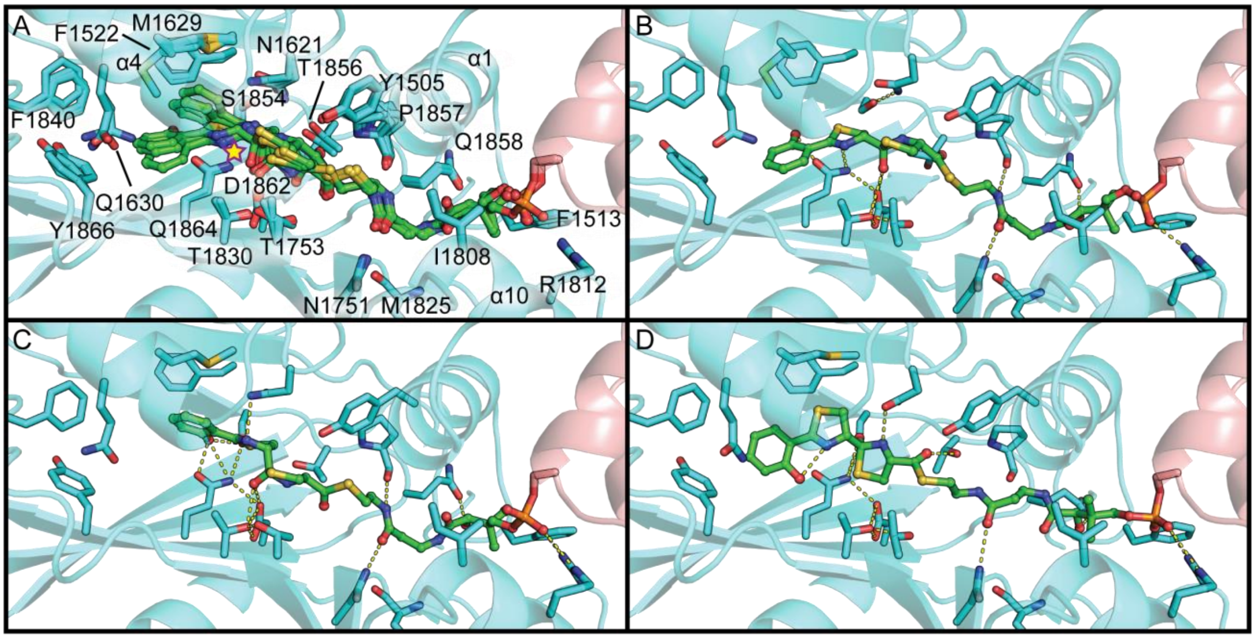

#	Panel	Dyad State	Glide Docking Score	Prime Energy kcal/mol	Prime MM-GBSA dG Bind kcal/mol	Complex Energy kcal/mol	Receptor Energy kcal/mol	Receptor Strain Energy kcal/mol	Ligand Energy kcal/mol	Ligand Strain Energy kcal/mol
7	B	negative	-8.5	-20935	-79	-21073	-20896	5.4	-98.1	18
8	C	negative	-10.0	-20906	-55	-21045	-20895	8.2	-94.9	9.1
9	D	negative	-8.8	-20925	-73	-21063	-20887	5.0	-92.2	12

Figure S15 – Ppant-2HPTT(*S*)-OH covalent docking poses in hydroxyl-toward-dyad or deeply placed hydroxyl-toward-N-terminal-sheet orientations. HMWP2-Cy2 is in cyan, and Ppant-2HPTT-OH is in green. **A**, A superimposition of the class of poses in which the leaving group oxygen is directed toward the putatively catalytic dyad (HMWP2 T1830-D1862) is shown. A yellow star marks the leaving group oxygen. This class positions the pantetheine thioester so that Y1505 could potentially interact with its sulfur. These poses consistently place the Ppant to allow hydrogen bonding with N1751 and typically also with the carbonyl of P1857. Only the 2HPT orientation with the phenol hydroxyl toward the thiazoline sulfur are observed. **B and C**, Representative top poses of the hydroxyl-toward-dyad orientation are displayed in the same order as in the table at the bottom of the figure (sorted by Prime Energy of the refined covalent docking complex). These poses position the Ppant dimethyl in states resembling either AB3403 (PDB ID 4ZXI) or ObiF (PDB ID 6N8E), and all necessitate a flip of the leaving group oxygen from toward the N-terminus of helix α4 (where it would be expected to reside following the condensation reaction) to toward the dyad, which would likely also necessitate substantial motion of the 2HPT side chain. **D**, This pose is an outlier of the hydroxyl-toward-N-terminal-sheet class placing the 2HPT side chain most deeply in the side chain-binding region and displays low divergence from 2HPT planarity in the orientation with the phenol hydroxyl toward the thiazoline nitrogen. The leaving group oxygen would have to undergo a large rotation from its hypothesized position during condensation to obtain this position during cyclodehydration, which would probably also necessitate similarly largescale motion of the 2HPT side chain. The table displays docking scores and energy properties for each pose in panels B-D. Table coloration uses the same scale in Figs. S13-18 and serves to highlight more favorable poses. Darker green corresponds to favorable energy properties. Darker red corresponds to less favorable strain energy terms.

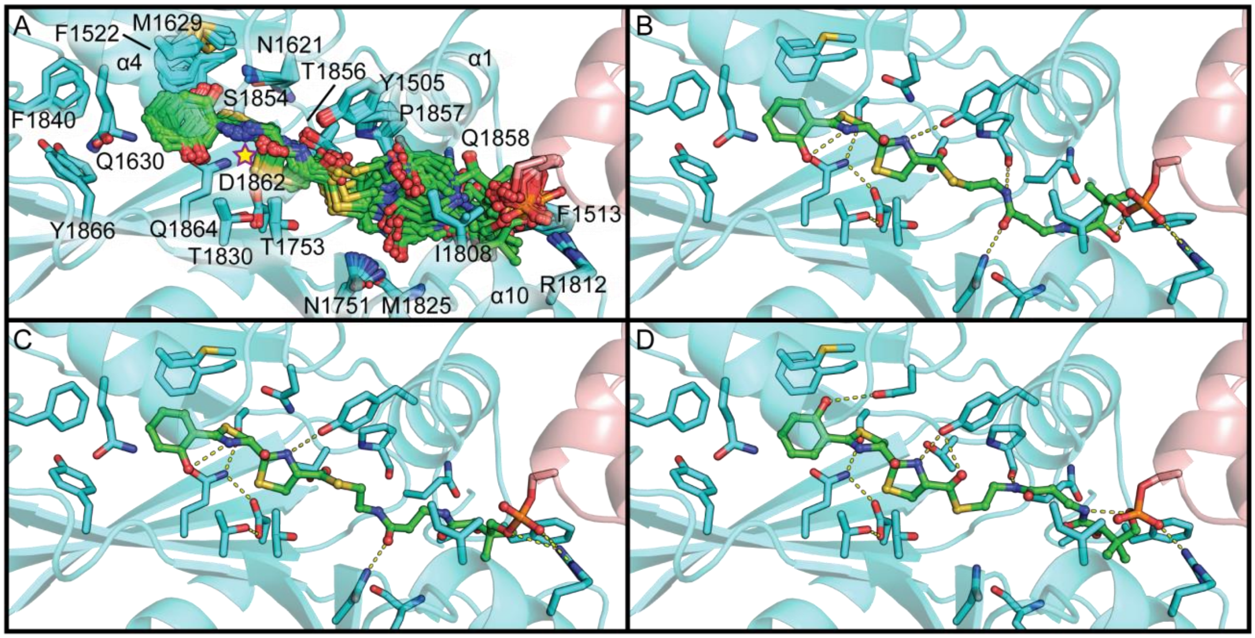

#	Panel	Dyad State	Glide Docking Score	Prime Energy kcal/mol	Prime MM-GBSA dG Bind kcal/mol	Complex Energy kcal/mol	Receptor Energy kcal/mol	Receptor Strain Energy kcal/mol	Ligand Energy kcal/mol	Ligand Strain Energy kcal/mol
10	B	neutral	-10.4	-20936	-82	-21083	-20904	6.1	-96.4	8.3
11	C	neutral	-10.0	-20935	-80	-21080	-20903	5.1	-96.4	9.1
12	D	neutral	-9.2	-20931	-64	-21069	-20913	10	-91.6	9.8

Figure S16 – Hydroxyl-toward-α4 Ppant-2HPTT(*R*)-OH poses from covalent docking. HMWP2-Cy2 is in cyan, and Ppant-2HPTT-OH is in green. **A**, A superposition of the class of poses in which the leaving group oxygen is directed toward helix α4 is shown. This class displays both 2HPT orientations and some degree of diversity at the pantetheine thioester linkage and the pantetheine amine position near the dimethyl group. Some poses place the dimethyl moiety of pantetheine toward I1808 in a manner similar to AB3403 (PDB ID 4ZXI), but most poses place it in the opposite direction in a way that resembles ObiF1 (PDB ID 6N8E) or between the two, with the dimethyl directed toward the loop after helix α10. Additionally, the pantetheine amide nearer the thioester linkage appears to orient either so its N-H forms a hydrogen bond with the carbonyl of P1857 or so its carbonyl oxygen can act as hydrogen bond acceptor to N1751—sometimes also allowing hydrogen bonding between its N-H and the carbonyl of P1857. As observed in some of the Ppant-2HPTT(*S*)-OH poses, the hydroxyl of Y1505 is in the vicinity of the pantetheine thioester linkage and/or the adjacent pantetheine amide, suggesting its ability to act as hydrogen bond partner with these groups. **B-D**, Representative top poses are displayed in the same order as in the table at the bottom of the figure (sorted by Prime Energy of the refined covalent docking complex). Table coloration uses the same scale in Figs. S13-18 and serves to highlight more favorable poses. Darker green corresponds to favorable energy properties. Darker red corresponds to less favorable strain energy terms.

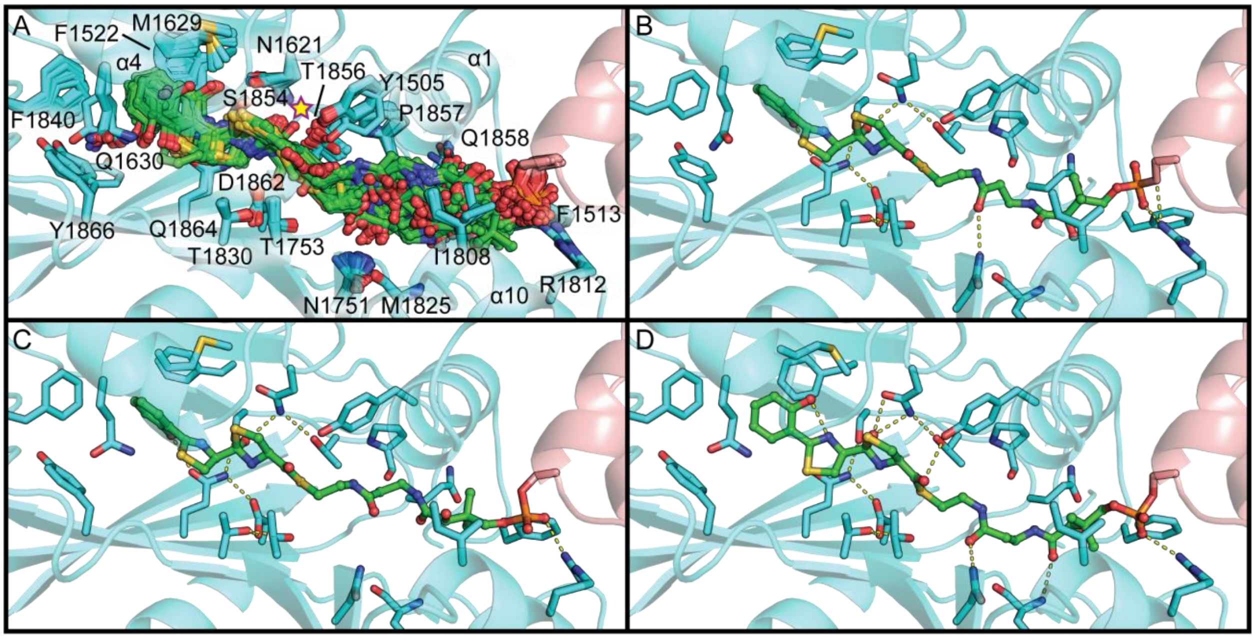

#	Panel	Dyad State	Glide Docking Score	Prime Energy kcal/mol	Prime MM-GBSA dG Bind kcal/mol	Complex Energy kcal/mol	Receptor Energy kcal/mol	Receptor Strain Energy kcal/mol	Ligand Energy kcal/mol	Ligand Strain Energy kcal/mol
13	B	neutral	-9.9	-20954	-73	-21098	-20923	6.7	-103	8.3
14	C	neutral	-10.4	-20951	-74	-21094	-20919	3.8	-102	6.4
15	D	neutral	-9.5	-20947	-76	-21096	-20920	6.7	-100	13

Figure S17 – Hydroxyl-toward-N-terminal-sheet Ppant-2HPTT(*R*)-OH poses from covalent docking. HMWP2-Cy2 is in cyan, and Ppant-2HPTT-OH is in green. **A**, A superposition of the class of poses in which the leaving group oxygen is directed toward the N-terminal β sheet is shown. This class displays a range of 2HPT and pantetheine conformations. Some poses place the dimethyl moiety of pantetheine toward I1808 in a manner similar to AB3403 (PDB ID 4ZXI), but most poses place it in the opposite direction in a way that resembles ObiF1 (PDB ID 6N8E) or between the positions, directed toward the loop after helix α10. Additionally, positioning of the pantetheine amides does not appear to particularly favor interactions with N1751 or P1857. Notably, however, Q1858 occupies several different rotameric states resembling EpoB-Cy/BmdB-Cy2 states. This allows for interactions between Q1858 and the pantetheine hydroxyl or the amide nearest to it. In these poses, the hydroxyl of Y1505 is in the vicinity of the carbonyl of the pantetheine thioester linkage. **B-D**, Representative top poses are displayed in the same order as in the table at the bottom of the figure (sorted by Prime Energy of the refined covalent docking complex). As noted for the other diastereomer's poses of this type, the leaving group oxygen would have to traverse a large distance from its expected position during the condensation reaction to obtain the positions observed in these poses. Table coloration uses the same scale in Figs. S13-18 and serves to highlight more favorable poses. Darker green corresponds to favorable energy properties. Darker red corresponds to less favorable strain energy terms.

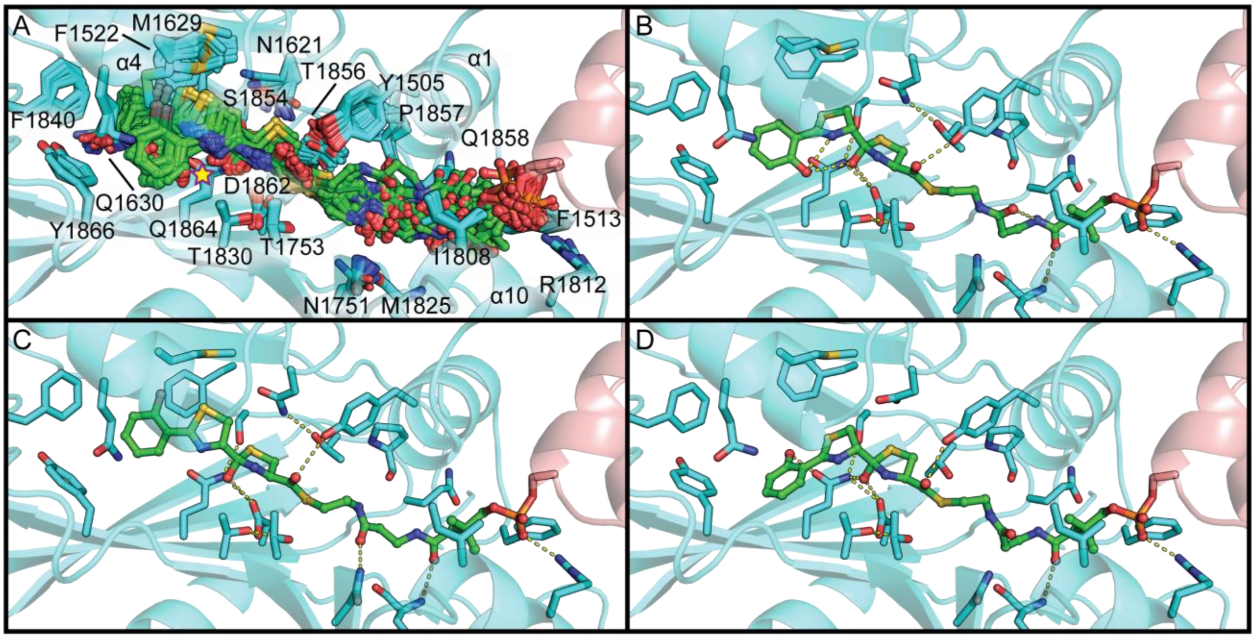

#	Panel	Dyad State	Glide Docking Score	Prime Energy kcal/mol	Prime MM-GBSA: dG Bind kcal/mol	Prime MM-GBSA: Complex Energy kcal/mol	Prime MM-GBSA: Receptor Energy kcal/mol	Prime MM-GBSA: Receptor Strain Energy kcal/mol	Prime MM-GBSA: Ligand Energy kcal/mol	Prime MM-GBSA: Ligand Strain Energy kcal/mol
16	B	neutral	-9.3	-20960	-72	-21104	-20927	8.1	-105	19
17	C	neutral	-9.4	-20954	-72	-21096	-20923	8.7	-100	14
18	D	neutral	-8.7	-20945	-77	-21087	-20913	2.5	-96.2	17

Figure S18 – Hydroxyl-toward-dyad Ppant-2HPTT(*R*)-OH poses from covalent docking. HMWP2-Cy2 is in cyan, and Ppant-2HPTT-OH is in green. **A**, A superposition of the class of poses in which the leaving group oxygen is directed toward the putatively catalytic dyad is shown. This class displays both 2HPT orientations and consistently permits interaction between Y1505 and the carbonyl of the pantetheine thioester linkage. Unlike the other classes of poses, many of these poses, including the class representatives, place the dimethyl moiety of pantetheine toward I1808 in a manner similar to AB3403 (PDB ID 4ZXI). Additionally, positioning of the pantetheine amides appears compatible with interactions with N1751 or the backbone N-H of M1825, and the amide nearest the pantetheine dimethyl moiety is consistently positioned in a way that is interesting when compared against alternative rotameric states of Q1858 observed in EpoB-Cy or BmdB-Cy2 (PDB IDs 5T7Z or 5T3E, respectively). **B-D**, Representative top poses are displayed in the same order as in the table at the bottom of the figure (sorted by Prime Energy of the refined covalent docking complex). As noted for the other diastereomer's poses of this type, the leaving group oxygen would have to traverse a large distance from its expected position during the condensation reaction to obtain the positions observed in these poses. Table coloration uses the same scale in Figs. S13-18 and serves to highlight more favorable poses. Darker green corresponds to favorable energy properties. Darker red corresponds to less favorable strain energy terms.

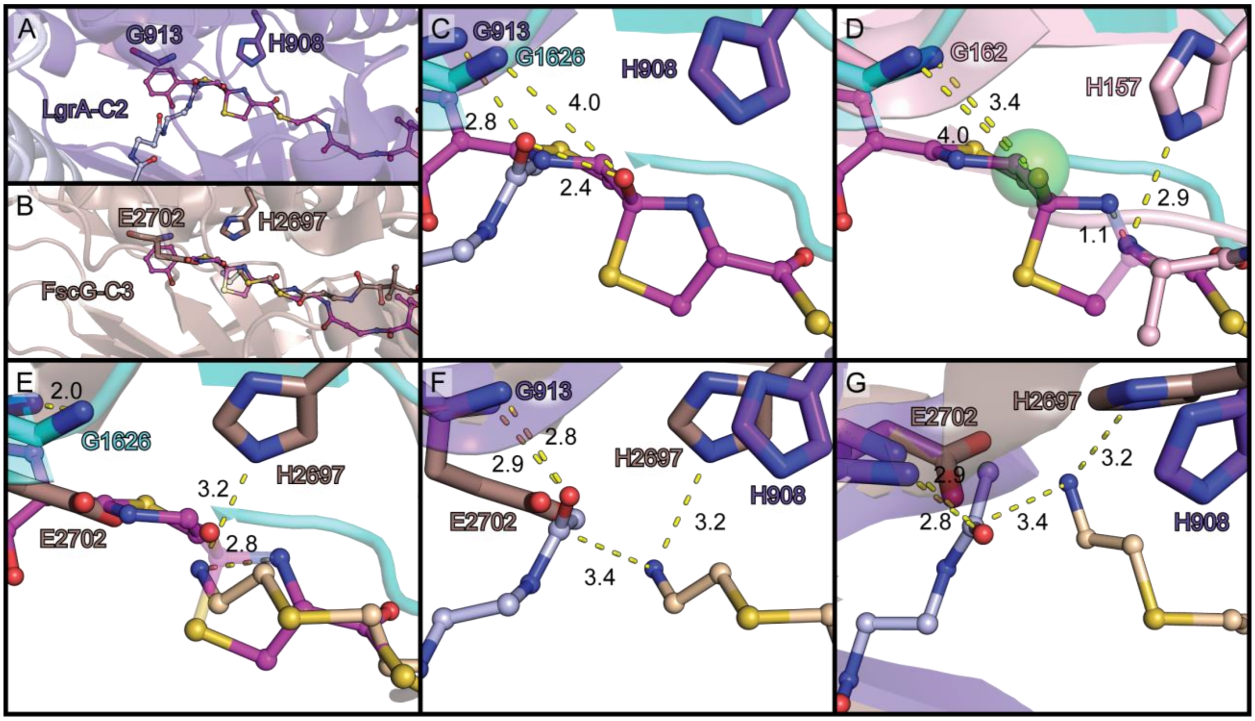

Figure S19 – Geometric rationale for a proposed pre-condensation state shared by C and Cy domains. A and B, These panels provide an overview of the active site regions of LgrA-C2 and FscG-C3, respectively. Coloration in these panels is continued through C, E, F and G. G913 and E2702 reside at the N-terminus of helix α4, and H908 is the conserved C domain histidine that may act as a base or to stabilize the nucleophile or zwitterionic, tetrahedral condensation transition state. The ball and stick representations are the ligands bound in LgrA-C2 (light blue) or FscG-C3 (wheat) superimposed with the top Ppant-2HPT(*R*)T-OH hydroxyl-toward-α4 pose (magenta). **C**, Displayed is the superimposition of the top Ppant-2HPT(*R*)T-OH hydroxyl-toward-α4 pose (magenta ball and stick and cyan protein) with LgrA-C2. The helix α4 N-terminal residue's backbone N-H in both systems is directed toward the leaving group oxygen. There is an approximately 1.2 Å increase in the distance between these groups going from HMWP2-Cy2 to LgrA-C2, but 2.4 Å separate the leaving group oxygen atoms in these models. **D**, Displayed is the superimposition of the top Ppant-2HPT(*R*)T-OH hydroxyl-toward-α4 pose (magenta ball and stick and cyan protein) with an acceptor substrate mimic-bound state of CDA-C1 (PDB ID 5DUA chain B, Bloudoff et al. 2016). This substrate mimic was found to be competent to undergo the condensation reaction to yield a tethered condensation product. Interestingly, in this active site at the N-terminus of helix α4, there is a chloride ion (green transparent sphere) that corresponds rather closely to the leaving group oxygen of the Ppant-2HPT(*R*)T-OH pose (~0.5 Å between centers). This superposition places the hydroxythiazolidine of the cyclized intermediate between analogous groups in the CDA-C1 model, with the nucleophilic nitrogen 1.1 Å from the hydroxythiazolidine nitrogen. Superposition of this CDA-C1 model with the LgrA-C2 model (not shown here) results in a slightly greater nucleophile-to-electrophile distance than that implied by the superimposition of LgrA-C2 and FscG-C3 in panels F and G. **E**, Displayed is the superimposition of the top Ppant-2HPT(*R*)T-OH hydroxyl-toward-α4 pose (magenta ball and stick and cyan protein) with FscG-C3 including a stabilized Ppant-glycine substrate mimic (wheat). The tetrahedral center bearing the leaving group oxygen of the cyclodehydration intermediate is shown in transparency so that the nucleophile position in FscG-C3 and the measurement between nitrogen atoms are visible. Notably, by interacting with E2702 the acceptor substrate mimic's terminal nitrogen is drawn closer to the N-terminus of helix α4 than in the CDA-C1 or HMWP2-Cy2 models. The position of E2702 is not commonly a large polar or charged residue, and the authors reporting the FscG-C3 structure note that it likely plays a role in positioning very small acceptor substrates such as glycinyl Ppant. **F and G**, Shown are views of the superposition of LgrA-C2 and FscG-C3 models related by a 90° rotation around the X axis. Most importantly, FscG E2702 occludes the path of the donor mimic in LgrA-C2. Additionally, the rotameric states of the conserved histidine residues in these models are rather different, demonstrating the ability of a residue at this position to rotate through a wide angle without largescale differences in surrounding residues. Although the nucleophile is relatively close to the electrophile in this superimposition, and their relative orientations form a favorable Bürgi-Dunitz angle (>90°), the nucleophile is offset from direct attack by a Flippin-Lodge angle around 45°.

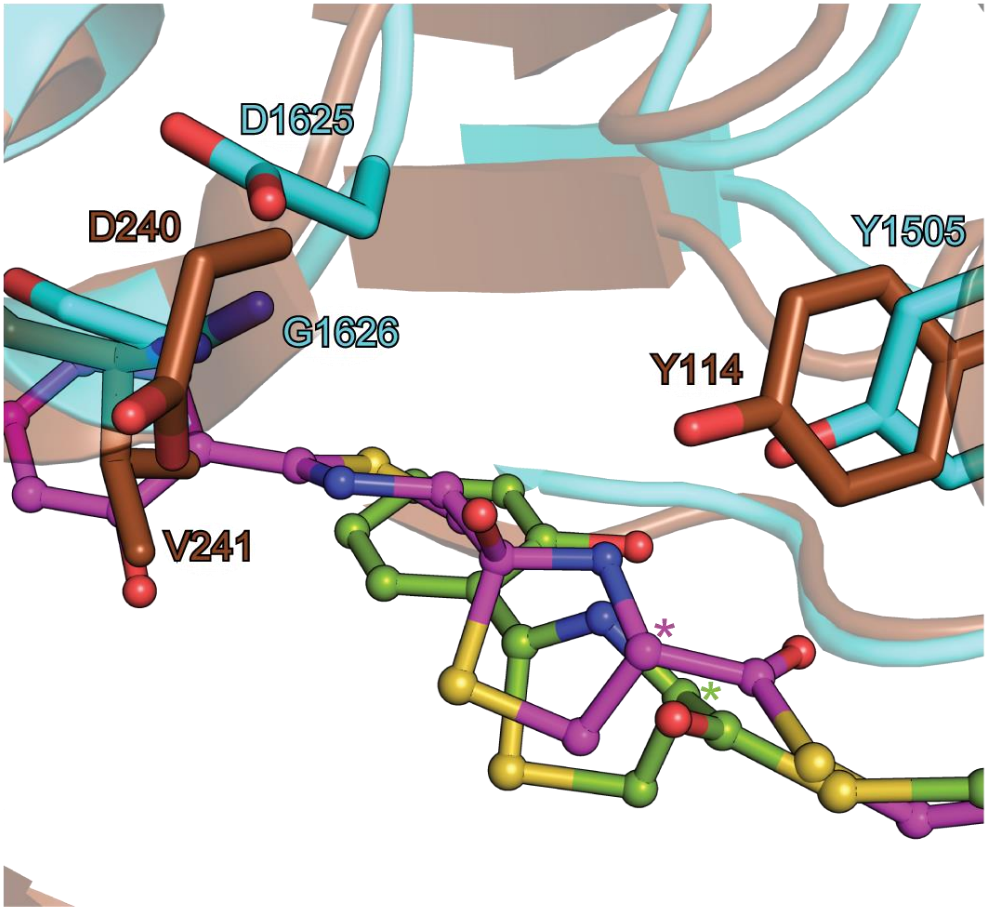

Figure S20 – Comparison of the top cyclodehydration intermediate pose from HMWP2-Cy2 and the product-bound state reported for PchE-Cy. The vicinities of the active sites differ between PchE-Cy (PDB ID 7EN1) and HMWP2-Cy2; however, the cyclodehydration intermediate docking model for HMWP2-Cy2 (magenta) is placed similarly to the product from PchE-Cy. Asterisks mark the Cα atoms derived from the acceptor cysteines from both models, which differ in chirality, with HMWP2-Cy2's corresponding to L-cysteine and PchE-Cy's corresponding to D-cysteine, as reported by Wang et al. (Wang et al., 2022).

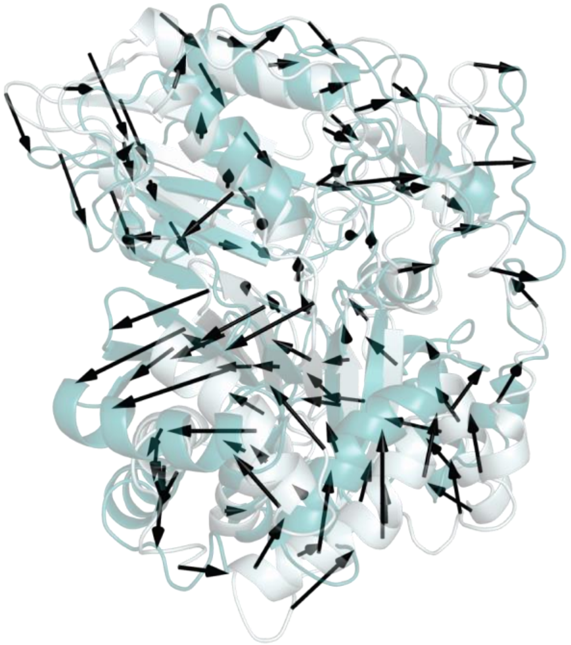

Figure S21 – The high-amplitude HMWP2-Cy2 low-frequency normal mode number 7. Mode 7 of HMWP2-Cy2 exhibits the greatest relative amplitude among the 10 lowest frequency modes. Here the extrema coordinates of the mode trajectory are displayed as pale cyan and deep teal, with distances between equivalent Cα atoms undergoing substantial displacement marked by black arrows. The left-most lobe of the C-terminal subdomain, comprised of strands 10, 12, 13 and 7, makes large movements away from the floor loop (larger vectors pointing toward the left side of the figure). With this motion, the tip of the N-terminal subdomain swings in toward the C-terminal subdomain. Consistent with comparisons against C domains in condensation donor-like states, part of the floor loop can also be seen to move away from the upstream tunnel entrance, toward the PCP1 linker and the hinge region between helices α5 and α6. (See also the movies of normal mode animations in the electronic supporting information.)

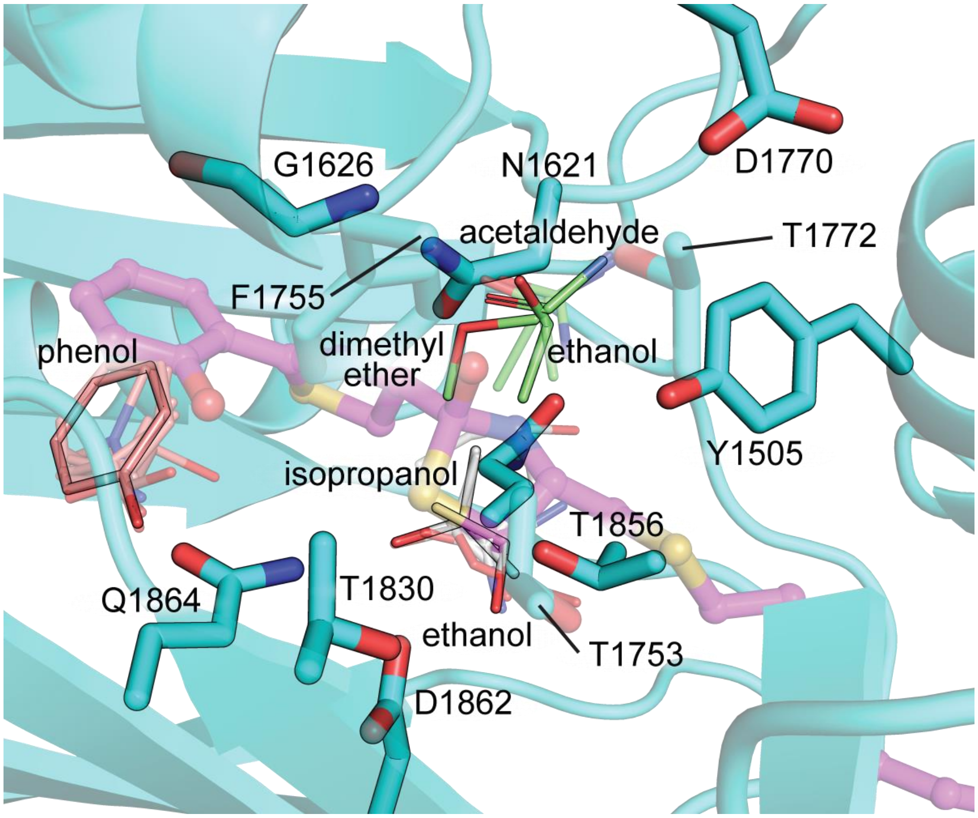

Figure S22 – The HMWP2-Cy2 active site binds small organic molecules in FTmap docking. Three clusters are displayed in salmon, white or light green thin sticks. Members of these clusters that are interesting in consideration of moieties of the cognate substrates are opaque, whereas other members of each cluster are transparent. Notably, hydroxyls of ethanol and isopropanol dock preferentially near the putatively catalytic dyad, oxygen atoms of ethanol and dimethyl ether are positioned at the N-terminus of helix α4 (G1626), loosely resembling crystallographic water sites, and phenol binds somewhat similarly to the hydroxyphenyl of Ppant-2HPT(*R*)T-OH in our top docking model (magenta ball and stick). Phenol is deeper into the side chain binding region than 2HPT of the docked pose, suggesting 2HPT may not be fully accessing the basin of attraction occupied by phenol in this volume. HMWP2-Cy2 residues from the floor loop (in the foreground) are displayed with transparency and are labeled with lines pointing to their Cα atoms.

References

Crooks, G.E., Hon, G., Chandonia, J., Brenner, S.E. (2004). WebLogo: A sequence logo generator. *Genome Res*. **14**, 1188-1190.

Bloudoff, K., Alonzo, D. A., and Schmeing, T. M. (2016) Chemical Probes Allow Structural Insight into the Condensation Reaction of Nonribosomal Peptide Synthetases. Cell Chem. Biol. **23**, 331-339.

Bloudoff, K., Fage, C. D., Marahiel, M. A., and Schmeing, T. M. (2017) Structural and mutational analysis of the nonribosomal peptide synthetase heterocyclization domain provides insight into catalysis. Proc. Natl. Acad. Sci. U. S. A. **114**, 95-100.

Dowling, D. P., Kung, Y., Croft, A. K., Taghizadeh, K., Kelly, W. L., Walsh, C. T., and Drennan, C. L. (2016) Structural elements of an NRPS cyclization domain and its intermodule docking domain. Proc. Natl. Acad. Sci. U. S. A. **113**, 12432-12437.

Drake, E. J., Miller, B. R., Shi, C., Tarrasch, J. T., Sundlov, J. A., Allen, C. L., Skiniotis, G., Aldrich, C. C., and Gulick, A. M. (2016) Structures of two distinct conformations of holo-non-ribosomal peptide synthetases. Nature **529**, 235-238.

Frishman, D., and Argos, P. (1995). Knowledge-based protein secondary structure assignment. *Proteins* **23**, 566-579.

Izoré, T., Candace Ho, Y. T., Kaczmarski, J. A., Gavriilidou, A., Chow, K. H., Steer, D. L., Goode, R. J. A., Schittenhelm, R. B., Tailhades, J., Tosin, M., Challis, G. L., Krenske, E. H., Ziemert, N., Jackson, C. J., and Cryle, M. J. (2021) Structures of a non-ribosomal peptide synthetase condensation domain suggest the basis of substrate selectivity. Nat. Commun. 12, 2511.

Koskinen, J.P. and Holm, L. (2012). SANS: high-throughput retrieval of protein sequences allowing 50% mismatches. *Bioinformatics* **28**, i438-i443.

Kreitler, D. F., Gemmell, E. M., Schaffer, J. E., Wencewicz, T. A., and Gulick, A. M. (2019) The structural basis of N-acyl-alpha-amino-beta-lactone formation catalyzed by a nonribosomal peptide synthetase. Nat. Commun. **10**, 3432.

Reimer, J. M., Eivaskhani, M., Harb, I., Guarné, A., Weigt, M., and Schmeing, T. M. (2019) Structures of a dimodular nonribosomal peptide synthetase reveal conformational flexibility. Science **366**, eaaw4388

Sievers, F., Wilm, A., Dineen, D., Gibson, T.J., Karplus, K., Li, W., Lopez, R., McWilliam, H., Remmert, M., Söding, J., Thompson, J.D. and Higgins, D.G. (2011). Fast, scalable generation of high-quality protein multiple sequence alignments using Clustal Omega. *Mol. Syst. Biol*. **7**, 539.

Somervuo, P., and Holm, L. (2015). SANSparallel: interactive homology search against Uniprot. *Nucl. Acids Res*. **43**, W24-W29.

Wang, J., Li, D., Chen, L., Cao, W., Kong, L., Zhang, W., Croll, T., Deng, Z., Liang, J., and Wang, Z. (2022). Catalytic trajectory of a dimeric nonribosomal peptide synthetase subunit with an inserted epimerase domain. *Nat. Commun*. **13**, 592.

Waterhouse, A.M., Procter, J.B., Martin, D.M.A., Clamp, M., Barton, G.J. (2009). Jalview Version 2—a multiple sequence alignment editor and analysis workbench. *Bioinformatics* **25**, 1189-1191.

Williams, C.J., Headd, J.J., Moriarty, N.W., Prisant, M.G., Videau, L.L., Deis, L.N., Verma, V., Keedy, D.A., Hintze, B.J., Chen, V.B., *et al.* (2018). MolProbity: More and better reference data for improved all-atom structure validation. *Protein Sci.* **27**, 293-315.
